# Differences in recovery time between trapeziectomy and carpometacarpal joint replacement: meta-analysis

**DOI:** 10.1093/bjsopen/zrag040

**Published:** 2026-04-29

**Authors:** Lauren Y Chong, Olivia J Hartrick, Chetan Khatri, Ciaran Sandhu, Sumedh Bele, Jeremy Rodrigues, Conrad Harrison

**Affiliations:** School of Medicine and Biomedical Sciences, University of Oxford, Oxford, UK; Healthcare Management Leadership Innovation, Oxford University Hospitals NHS Trust, Oxford, UK; Stoke Mandeville Hospital, Buckinghamshire Healthcare Trust, Buckinghamshire, UK; Clinical Trials Unit, University of Warwick, Coventry, UK; School of Medicine and Biomedical Sciences, University of Oxford, Oxford, UK; Nuffield Department of Orthopaedics, Rheumatology and Musculoskeletal Sciences, University of Oxford, Oxford, UK; Clinical Trials Unit, University of Warwick, Coventry, UK; Nuffield Department of Orthopaedics, Rheumatology and Musculoskeletal Sciences, University of Oxford, Oxford, UK

**Keywords:** thumb-base osteoarthritis, hand function, post-operative recovery

## Abstract

**Background:**

Thumb-base osteoarthritis is a common degenerative condition that produces symptoms including pain and reduced hand function. Trapeziectomy is the mainstay surgical intervention following failure of conservative management and steroid injection, with carpometacarpal (CMC) joint replacement anticipating faster recovery time and return to work. However, there is limited evidence on recovery following both surgeries, which affects patient counselling and future research design.

**Methods:**

A preregistered (CRD42023450865) systematic review of the literature was conducted according to the PRISMA guidelines. Disability of the Arm, Shoulder and Hand (DASH) questionnaire scores, grip strength, and key pinch strength scores were obtained at different timepoints during follow-up. Standardized mean changes (SMC) were calculated and pooled through random-effects meta-analysis before being plotted at monthly timepoints.

**Results:**

Of 63 articles eligible for inclusion, 25 studies were included in the meta-analysis. Subgroup analyses were conducted comparing types of trapeziectomy, joint replacement implants, and immobilization protocols. At the 1-month follow-up, joint replacement was associated with a trend of improvement in the DASH score, whereas trapeziectomy was associated with a deterioration. At 12 months, joint replacement showed a trend of greater improvement in hand function than trapeziectomy. Key pinch strength returned to baseline function at 1.5 months after joint replacement, compared with 6 months after trapeziectomy. Newer dual-mobility implants were associated with a quicker trend of recovery in DASH and key pinch strength than single-mobility implants. However, confidence intervals overlapped considerably, and most study arms had a high risk of bias, so these findings should not be considered statistically significant.

**Conclusion:**

This study shows a trend towards faster early recovery after CMC joint replacement compared with trapeziectomy, although the evidence remains limited. Randomized clinical trials with systems to frequently capture patient-reported outcomes data are required to study the temporal course of recovery for both surgeries.

## Teaser

This study aimed to define and compare patient-reported recovery trajectories following trapeziectomy and carpometacarpal (CMC) joint replacement for thumb-base osteoarthritis. This study provides level II evidence that CMC joint replacement results in faster recovery than trapeziectomy over the first 3 months after surgery, with newer dual-mobility implants associated with quicker recovery in DASH and key pinch strength than single-mobility implants. These findings can be used to counsel patients in their recovery and to aid in future study designs.

## Introduction

Thumb-base osteoarthritis is common and has a propensity to affect postmenopausal women aged > 50 years^1^. Cartilage loss and bony impingement of the carpometacarpal (CMC) joint are associated with pain and reduced hand function, resulting in a large burden of disease by impacting day-to-day activities and having a negative psychological impact^2,3^. Conservative management is most effective for early (Eaton stage 1) disease, which includes splints and slings for joint stability, and analgesia and intra-articular steroid injections for pain relief^1^. Following failure of these therapies, surgical intervention is indicated for treating persistent pain and hand dysfunction, for which trapeziectomy with or without ligament reconstruction and tendon interposition (LRTI) are the mainstay practice^4^.

CMC joint replacement for the treatment of thumb-base osteoarthritis is increasingly popular, with proponents arguing that it yields a more rapid recovery than trapeziectomy, resulting in a faster return to work and daily activities^5^. Newer dual-mobility implants for CMC joint replacement are purported to provide greater joint stability and lower dislocation rates than single-mobility implants^6^. Recent systematic reviews have suggested equivalent and potentially superior outcomes for CMC joint replacement compared with trapeziectomy with respect to pain relief and restoration of hand function^7^. However, the evidence largely relies on cross-sectional endpoints, which are limited because they do not indicate the recovery trajectory over time. The various trapeziectomy techniques and joint replacement implants used in practice also complicate comparisons between interventions^8^. As such, inconsistencies have been reported in the peer-reviewed literature and patient information regarding recovery times^9–11^. This knowledge gap affects treatment selection and poses challenges in selecting appropriate endpoints for clinical research.

In many cases, CMC joint replacement is associated with greater technical demands, costs, and complications than trapeziectomy^12^. Long-term analyses into the economics of CMC joint replacement *versus* trapeziectomy are underway, where various factors, including implant survival, revision rates, time-to-return to function, and quality-adjusted life-years gains, may offset an initial implant cost^13,14^. In the meantime, evaluation of the purported comparative benefits of CMC joint replacement, such as enhanced recovery, will help clinicians to contextualize these costs.

Recovery can be measured over time with patient-reported outcome measures (PROMs) such as the Disability of the Arm, Shoulder, and Hand (DASH) questionnaire^15^. These measures aim to capture symptom severity from the patient's perspective. When plotting PROM scores over time, it is possible to see an improvement in symptoms following intervention (a negative gradient in the case of the DASH score) and a plateau in scores if the patient enters a stable recovered state. By capturing serial PROM measurements, recovery can be compared (for example, by time-to-plateau, recovery gradient, area under the curve, or by comparing symptom severity at specific timepoints). Similar analyses can be conducted with clinician-reported outcome measures, such as grip and pinch strengths. This would aid in counselling patients and providing more robust information for future trial design (for example, trial endpoints). Recent trajectory-based meta-analyses have demonstrated how modelling change over time can reveal clinically relevant patterns that are missed when relying solely on infrequent cross-sectional endpoint comparisons^16^.

The aim of this study was to describe and compare patient and clinician-reported recovery trajectories for trapeziectomy with or without LRTI and CMC joint replacement, through systematic review and meta-analysis.

## Methods

This systematic review was conducted according to the PRISMA guidelines. The study protocol was registered in the PROSPERO database (CRD42023450865).

Studies were included if they reported any PROM at prospectively defined timepoints, collected within 1 year of surgical intervention. Studies were limited to English-language publications.

Studies with patients under 18 years of age, revisional/secondary trapeziectomy or joint replacement surgeries, partial trapeziectomies, and animal or cadaveric studies were excluded. If data were missing, unreported, partially reported, or reported in a way that did not allow for comparison, up to two attempts were made to contact the corresponding author before excluding the study.

A literature search was conducted on December 2025, using MEDLINE, EMBASE, Cumulative Index to Nursing and Allied Health Literature (CINAHL), ISI Web of Science, and the Cochrane Central Register of Controlled Trials. Search strategies are provided in *Tables S1–S5*.

Two authors (L.Y.C., S.B.) used Covidence (Veritas Health Innovation Ltd, Melbourne, Australia) to review and remove duplicates, followed by title, abstract and full text screening. Each paper was screened independently according to the inclusion and exclusion criteria, and conflicts were resolved during meetings. Any disagreements with inclusion or exclusion were resolved through discussion with a senior author (C.H.).

### Data collection

The authors independently extracted data from each study (L.Y.C., O.J.H.). The corresponding authors of the studies were contacted if the study data were not represented in numerical format or if information was missing. If no response was received or there was further uncertainty, the study was excluded.

Data from each study arm were extracted independently (that is, if a comparative study had a two-arm design, data were extracted from each arm separately). The data extracted included the number of patients, mean patient age, sex ratio (male : female), intervention type (trapeziectomy or CMC joint replacement), type of trapeziectomy (simple trapeziectomy, LRTI, suspension arthroplasty, or interposition arthroplasty), joint replacement implant type (dual-mobility, single-mobility, cemented, or uncemented implants), and immobilization protocol with a cast, thumb spica, or K-wire fixation (< 1 week, 1–2 weeks, 2–4 weeks, or > 4 weeks). In each study, the postoperative timepoints and outcome measures used for data collection were recorded.

### Bias assessment

In this study, bias was defined as error that may cause the mean PROM or strength score at a specific timepoint to appear higher or lower than the true population mean. That is, bias may cause a misrepresentative recovery trajectory in a given cohort (study arm). Two authors worked independently and in duplicate (L.Y.C., C.S.) using a modified version of the National Institutes of Health’s Quality Assessment Tool for Before-After (Pre-Post) Studies With No Control Group criteria^17^ to assess the bias of each study arm independently. If there were discrepancies, the risk of bias scores for each study arm were discussed until an agreement was reached. This allowed for relevant bias analyses (affecting the recovery trajectory plot) in each study arm (that is, each cohort of patients receiving a given intervention), separately.

### Outcomes of interest

The most commonly reported PROM was the DASH score, which served as the primary outcome for the quantitative synthesis. Secondary outcome measures included grip strength and key pinch strength, both measured in kilograms.

The recovery for each study arm was assessed by calculating the bias-corrected standardized mean change (SMC) in DASH scores at each timepoint. This represents the study arm’s mean symptomatic improvement, relative to baseline, at each timepoint. The SMC scores were calculated by subtracting the mean score at follow-up from the mean baseline score, dividing it by the baseline standard deviation, and multiplying it by a bias correction factor based on group size^18,19^. An effect size of 0.3–0.5 is used to approximately represent the minimal clinically meaningful difference, in the absence of more robust estimates of minimal important differences^20^. If the pooled standard deviation was not reported, the baseline or follow-up standard deviation was used. This analysis was repeated for grip strength and key pinch strength.

### Data analysis

SMC scores were pooled for each intervention through random effects meta-analysis^21^. Meta-analyses were conducted of SMC scores recorded, as the mean and standard deviation, at 1, 3, 6, and 12 months (that is, an individual meta-analysis of SMC in DASH score was performed for each procedure, at each timepoint; this was then repeated for grip strength and key pinch strength). The pooled SMC estimates are presented from each meta-analysis with 95% confidence intervals as time series plots.

Subgroups analyses were performed for cemented *versus* uncemented implants, dual-mobility *versus* single-mobility implants, types of trapeziectomy, and immobilization regimens. Cemented implants included Elektra cemented DLC all-polyethylene screw cup, whereas uncemented implants included TOUCH® TMC joint press-fit conical cones, Elektra press-fit stem uncemented screwed cup, MAIA™ prosthesis, and CARPOFIT® TMC prosthesis (manufacturer details available in *Table S6*). Dual-mobility implants included TOUCH® TMC joint press-fit conical cones and MAIA™ prosthesis, whereas single-mobility implants included the Elektra cemented and non-cemented screw cups. The different types of trapeziectomy included simple trapeziectomy, LRTI, and suspension arthroplasty, including Epping and TightRope with no interposition and interposition arthroplasty (for example, with the use of silicone spacers). Immobilization regimens were determined by the use of a cast, thumb spica, or K-wires, and were categorised as < 1, 1–2, 2–4, and > 4 weeks.

## Results

### Study selection

**Fig. 1:**
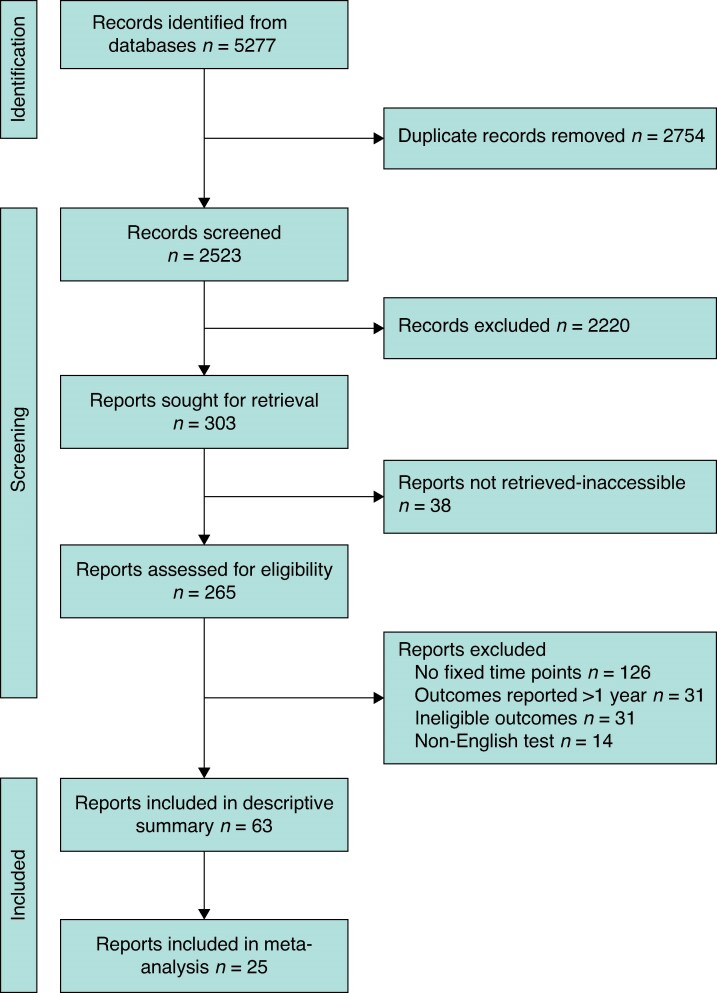
Study selection according to the PRISMA flow diagram Databases and registries were searched on 19 December 2025.

In all, 2523 unique articles were identified from the literature search after duplicates had been removed. Overall, 265 full texts were screened for eligibility, with 63 studies meeting the inclusion criteria (*Fig. 1*)^5,22–83^. Of the 63 studies included, there were 12 randomized clinical trials and 51 prospective cohort studies. In all, 5806 participants underwent trapeziectomy and 892 underwent CMC joint replacement. The mean age across studies was 59.6 years and the male to female ratio was 0.31 (*Table S6*). Of these 63 studies, 25 studies (37 study arms) reported data that could be meta-analysed^5,22–25,36,39,41–43,48,49,51,53,56,59,63,66,67,72,74,75,80,81,83^.

### Risk of bias

The 37 study arms were analysed individually. Thirteen study arms had a moderate risk of bias and 24 study arms had a high risk of bias (*Table S7*).

### Meta-analysis

Overall, 935 patients who underwent trapeziectomy and 235 who underwent CMC joint replacement were included in the meta-analysis. The mean age was 59.8 years. The male-to-female ratio was 0.28^5,22–25,36,39,41–43,48,49,51,53,56,59,63,66,67,72,74,75,80,81,83^.

*Figures 2–4* illustrate the pooled recovery trajectories for each procedure, combining meta-analyses at each time point, whereas forest plots for each meta-analysis at different timepoints are provided in *Figs S1–S25*.

**Fig. 2:**
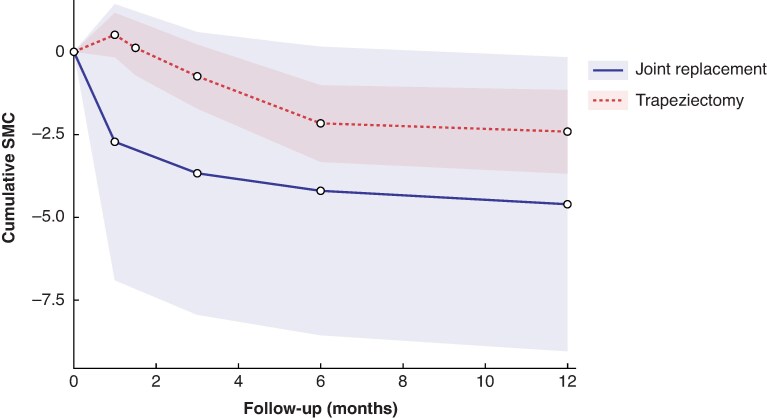
Cumulative SMC in Disability of the Arm, Shoulder and Hand scores for trapeziectomy and CMC joint replacement Each point represents a pooled SMC estimate from an individual meta-analysis. Not every study contributed data to each point. A lower score indicates better clinical outcomes. Shaded areas represent 95% confidence intervals. SMC, standardized mean change; CMC, carpometacarpal.

**Fig. 3:**
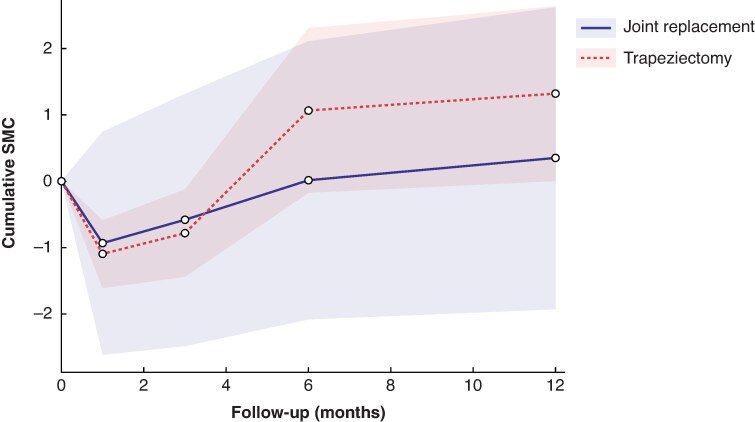
Cumulative SMC of grip strengths for trapeziectomy and CMC joint replacement Each point represents a pooled SMC estimate from an individual meta-analysis. Not every study contributed data to each point. A higher score suggests better clinical outcomes. Shaded areas represent 95% confidence intervals. SMC, standardized mean change; CMC, carpometacarpal.

**Fig. 4:**
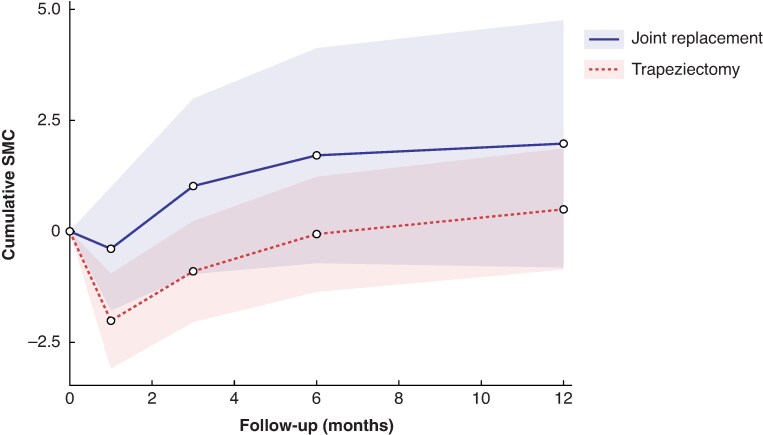
Cumulative SMC of key pinch strengths for trapeziectomy and CMC joint replacement Each point represents a pooled SMC estimate from an individual meta-analysis. Not every study contributed data to each point. A higher score suggests better clinical outcomes. Shaded areas represent 95% confidence intervals. SMC, standardized mean change; CMC, carpometacarpal.

*Figure 2* shows SMCs in DASH scores for trapeziectomy and joint replacement, demonstrated by the results of nine different meta-analyses (*Figs S1–S9*), one for each intervention at each timepoint. *Figure 2* shows that joint replacement was associated with an improvement in the DASH score at the 1-month follow-up, whereas trapeziectomy was associated with a deterioration in the DASH score over the same period. By 3 months, patients undergoing either intervention showed improvements in DASH scores. At 12 months, joint replacement resulted in a greater improvement in hand function compared with trapeziectomy (*Fig. 2*).

*Figure 3* shows SMCs in grip strength for trapeziectomy and joint replacement, demonstrated by the results of eight different meta-analyses (*Figs S10–S17*). *Figure 3* shows that grip strength deteriorated in patients who underwent trapeziectomy compared with joint replacement at 1 and 3 months. By 6 months, both interventions showed an improvement in grip strength, with trapeziectomy showing greater improvement than joint replacement at 12 months (*Fig. 3*).

*Figure 4* shows SMCs in key pinch strength for trapeziectomy and joint replacement, demonstrated by the results of eight different meta-analyses (*Figs S18–S25*). *Figure 4* shows that patients who underwent trapeziectomy had a more significant deterioration in key pinch strength at 1 month than patients who underwent joint replacement. Both interventions showed an improvement in key pinch strength by 6 months. However, key pinch strength took 6 months to return to baseline in the trapeziectomy group, compared with 1.5 months in the joint replacement group (*Fig. 4*).

**Fig. 5:**
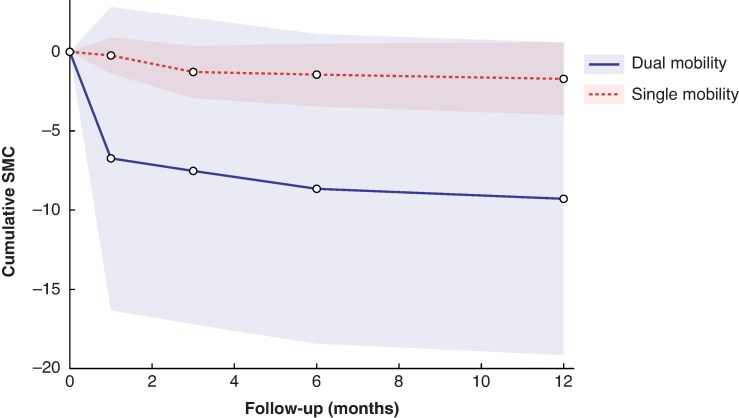
Cumulative SMC in Disability of the Arm, Shoulder and Hand scores for dual mobility *versus* single mobility CMC joint replacement SMC, standardized mean change; CMC, carpometacarpal.

The pooled recovery trajectories for subgroups analyses for each outcome measure were reported as follows: uncemented joint replacement (*Figs S26–S28*), dual-mobility *versus* single-mobility implants (*Fig. 5* and *Figs S29*, *S30*), types of trapeziectomy (*Figs S31–S33*), and immobilization regimens (*Figs S34–S36*). In the subgroup analyses, a quicker recovery of DASH scores and key pinch strength was documented for dual-mobility *versus* single-mobility implants, with no significant difference in grip strength recovery (*Fig. 5* and *Figs S29*, *S30*). For the different immobilization regimens and types of trapeziectomy, no significant differences were found in DASH scores, grip strength, and key pinch strength recovery (*Figs S34–S36*).

In each figure, confidence intervals between the recovery trajectories of each intervention were broad and overlapped considerably, so these findings should not be considered statistically significant.

## Discussion

This study aimed to describe and compare patient-reported recovery trajectories following trapeziectomy with or without LRTI and CMC joint replacement, based on meta-analyses of published literature. The findings suggest a trend towards faster and earlier recovery following CMC joint replacement compared with trapeziectomy, with the use of newer dual-mobility implants appearing to result in quicker recovery in DASH and key pinch strength than single-mobility implants. No convincing differences were documented between the type of trapeziectomy or immobilization regimen. Given the considerable heterogeneity, overlapping confidence intervals, and generally high risk of bias across included studies, this information should be interpreted cautiously. Nonetheless, these results may help better inform the design of future, higher-quality studies.

There are important limitations to note when interpreting the results of this study. First, this study presents level II evidence: patients were not randomized to receive either trapeziectomy or CMC joint replacement. Second, the studies included are clinically heterogeneous, with a range of techniques performed, and there is considerable confidence interval overlap between recovery trajectories for each intervention. Furthermore, the subgroup analyses were exploratory in nature and limited by small sample sizes within each comparison, rendering them underpowered to detect modest but potentially important effects. Consequently, these findings should be interpreted as hypothesis generating rather than hypothesis testing, warranting confirmation in larger, well-powered studies. Third, the risk of bias (that is, the risk that individual study scores are higher or lower than the true population score would be at a given timepoint) was generally high. A comparative risk of bias tool, such as Cochrane RoB2 tool or ROBINS-I, was not used because these assess the risk of bias favouring one study arm over another and assume all studies have multiple arms. Finally, the data have been interpolated linearly between timepoints (whereas, in real life, the trajectories of these groups may not follow straight lines).

Despite these limitations, the results of the present study show a consistent signal between each outcome. They are also supported by published data not included in the present meta-analyses. For example, Klim *et al*.^45^ showed better pain (visual analogue scale) and quality of life (Short-Form McGill Pain Questionnaire) scores at 6 weeks in their joint replacement group than in their Epping resection-suspension arthroplasty group, but similar scores in each group by 12 months. De Jong *et al*.^36^ showed better patient satisfaction and Mental Health Quotient (MHOQ) at 3 months in their joint replacement group compared with their trapeziectomy group, but similar scores at 12 months.

Similarly, another author^84^ reported a median return-to-work time of 3 months following surgical intervention for CMC joint osteoarthritis, whereas others reported a mean return-to-work time of 48 days with the use of a CMC joint replacement^85^. Time taken to return to work is a cruder indicator of recovery rate than PROM trajectory, because it is often reported retrospectively, is influenced by the nature of the patients’ work (among other factors), and many patients with CMC joint osteoarthritis are not working.

Complication rates can impact recovery trajectories and may cause a patient to follow a very different path to the group-level trajectories reported here. Trapeziectomy complications include subsidence, weakness, and persistent pain, whereas joint replacement complications include joint loosening, dislocation, and implant failure^84,86,87^. A systematic review reported severe complications (including thumb collapse and metacarpophalangeal hyperextension) in 6% of trapeziectomies and a 2% revision rate, compared with a 24% rate of severe complications following CMC joint replacement surgeries (including loosening, dislocation, and wear) and a 13% revision rate^86^. These estimates are potentially affected by the biases prevalent in observational data and, as joint replacements evolve, one could expect a reduction in the complication rate. The comparative safety of these interventions will become clearer as randomized clinical trials emerge.

Future trials should consider high-frequency data capture in the first 3 months after surgery to capture the largest differences in these groups, and endpoints at 12 months or later should be considered to capture the recovery plateaus. The Surgery *versus* Conservative Osteoarthritis of Thumb Trial (SCOOTT) is a randomized clinical trial funded by the National Institute for Health and Care Research to assess the cost and clinical effectiveness of thumb-base osteoarthritis treatment^88^. SCOOTT will compare the outcomes of physiotherapy, trapeziectomy, and CMC joint arthroplasty at 12 months using the Australian/Canadian Osteoarthritis Hand Index (AUSCAN™). Data from this trial could certainly aid front-line clinical and commissioning decisions surrounding the comparative effectiveness and safety of trapeziectomy and CMC joint arthroplasty, but the trial’s primary focus is not on early recovery trajectories. The Ecological Momentary Computerised Adaptive Testing (EMCAT) tool, which permits high-frequency PROM capture through burden-reducing adaptive algorithms, could help define recovery trajectories in future, and is currently being deployed in a (non-randomized) cohort study that will examine recovery in both trapeziectomy and CMC joint replacement^89–91^.

## Supplementary Material

zrag040_Supplementary_Data

## Data Availability

The data are available at the following repository: https://github.com/LCYE3005/Differences-in-recovery-time-between-trapeziectomy-and-CMC-joint-replacement.git
