# Supplementary material for: Differences in recovery time between trapeziectomy and carpometacarpal joint replacement: meta-analysis
Source: BJS Open. 2026 Apr 29;10(2):zrag040. doi: 10.1093/bjsopen/zrag040 (PMC13126664; doi:10.1093/bjsopen/zrag040)

**Differences in recovery time between trapeziectomy and carpometacarpal joint replacement: a systematic review and meta-analysis**

Miss Lauren Chong; BSc; School of Medicine and Biomedical Sciences, University of Oxford

Ms Olivia Hartrick; BMBS, MSc Healthcare management leadership innovation; Oxford University Hospitals NHS Trust; [o.hartrick@nhs.net](mailto:o.hartrick@nhs.net)

Mr Chetan Khatri; PhD, MRCS(Eng); Clinical Trials Unit, University of Warwick; [chetan.khatri@gmail.com](mailto:chetan.khatri@gmail.com)

Mr Ciaran Sandhu; BA (Oxon); School of Medicine and Biomedical Sciences, University of Oxford; [ciaran.sandhu@queens.ox.ac.uk](mailto:ciaran.sandhu@queens.ox.ac.uk)

Dr Sumedh Bele; MBBS, PhD; SITU, NDORMS; [sumedh.bele@ndorms.ox.ac.uk](mailto:sumedh.bele@ndorms.ox.ac.uk)

Professor Jeremy Rodrigues; PhD, FRCS(Plast); Clinical Trials Unit, University of Warwick; [j.rodrigues@warwick.ac.uk](mailto:j.rodrigues@warwick.ac.uk)

Mr Conrad Harrison; DPhil; Nuffield Department of Orthopaedics, Rheumatology and Musculoskeletal Sciences, University of Oxford; [conrad.harrison@ndorms.ox.ac.uk](mailto:conrad.harrison@ndorms.ox.ac.uk)

Corresponding author. Mr Conrad Harrison; NDORMS, University of Oxford; [conrad.harrison@ndorms.ox.ac.uk](mailto:conrad.harrison@ndorms.ox.ac.uk)

ORCID ID: https://orcid.org/0009-0000-9572-1561

**Supplementary Figures and Tables**

| **Table/Figure name** | **Page(s)** |
| --- | --- |
| Table S1-S5. Search strategy on MEDLINE, EMBASE, CINAHL, ISI Web of Science and Cochrane Central Register of Controlled Trials. | **2-6** |
| Table S6. Summary of characteristics of included studies | **7-32** |
| Table S7. Risk of bias assessment of included studies | **33-39** |
| Figures S1-25. Forest plots of DASH, grip strength and key pinch strength for trapeziectomy and joint replacement at 1, 3, 6 and 12 months | **40-52** |
| Figure S26-36. Cumulative SMC sub-group analyses | **53-59** |

Table S1. Search strategy on MEDLINE.

Date searched: 19/12/25; 1,560 results

| **#** | **Query** | **Results** |
| --- | --- | --- |
| 1 | osteoarthritis/ | 48,333 |
| 2 | osteoarthritis.ti,ab,kf. | 106,218 |
| 3 | 1 or 2 | 121,477 |
| 4 | thumb/ | 10,465 |
| 5 | trapezium bone/ | 801 |
| 6 | Carpometacarpal Joints/ | 1,386 |
| 7 | (thumb or thumbs or trapezium or trapezia or trapeziums or carpometacarpal*).ti,ab,kf. | 23,074 |
| 8 | 4 or 5 or 6 or 7 | 26,080 |
| 9 | arthroplasty, replacement/ | 7,111 |
| 10 | Joint Prosthesis/ | 11,063 |
| 11 | (arthroplast* or prosthes* or prosthet* or replac* or surger* or procedur* or operation*).ti,ab,kf. | 3,932,930 |
| 12 | 9 or 10 or 11 | 3,935,567 |
| 13 | 8 and 12 | 7,094 |
| 14 | trapeziectom*.ti,ab,kf. | 675 |
| 15 | arthroplasty, replacement, finger/ | 253 |
| 16 | 14 or 15 | 917 |
| 17 | 13 or 16 | 7,433 |
| 18 | 17 and 3 | 1,560 |

Table S2. Search strategy on EMBASE.

Date searched: 19/12/25; 1,465 results

| **#** | **Query** | **Results** |
| --- | --- | --- |
| 1 | Hand osteoarthritis/ | 2,824 |
| 2 | osteoarthritis.ti,ab,kf. | 154,915 |
| 3 | 1 or 2 | 155,152 |
| 4 | Thumb/ or thumb reconstruction/ | 14,310 |
| 5 | Carpometacarpal Joints/ | 2,849 |
| 6 | (thumb or thumbs or trapezium or trapezia or trapeziums or carpometacarpal*).ti,ab,kf. | 28,407 |
| 7 | Trapezium Bone/ | 795 |
| 8 | 4 or 5 or 6 or 7 | 31,463 |
| 9 | Replacement arthroplasty/ | 3,888 |
| 10 | Joint Prosthesis/ | 12,646 |
| 11 | (arthroplast* or prosthes* or prosthet* or replac* or surger* or procedur* or operation*).ti,ab,kf. | 5,334,845 |
| 12 | 9 or 10 or 11 | 5,337,516 |
| 13 | 8 and 12 | 9,175 |
| 14 | trapeziectom*.ti,ab,kf. | 794 |
| 15 | Finger replacement/ or thumb joint prosthesis/ | 151 |
| 16 | 14 or 15 | 921 |
| 17 | 13 or 16 | 9,424 |
| 18 | 17 and 3 | 1,465 |

Table S3. Search strategy on CINAHL.

Date searched: 19/12/25; 246 results

| # | Query | Results |
| --- | --- | --- |
| S1 | Osteoarthritis | 51318 |
| S2 | MH “Osteoarthritis, Hand” | 115 |
| S3 | S1 OR S2 | 51318 |
| S4 | MH “Thumb” | 1520 |
| S5 | MH “Carpometacarpal Joints” | 382 |
| S6 | Thumb OR thumbs OR trapezium OR trapezia OR trapeziums OR carpometacarpal* | 4543 |
| S7 | S4 OR S5 OR S6 | 4543 |
| S8 | MH “Arthroplasty, Replacement” | 4403 |
| S9 | MH “Joint Prosthesis” | 12511 |
| S10 | Arthroplast* OR prosthe* OR replac* OR surger* OR procedur* OR operation* | 1028374 |
| S11 | S8 OR S9 or S10 | 1028374 |
| S12 | S7 AND S11 | 1411 |
| S13 | Trapeziectom* | 108 |
| S14 | S12 OR S13 | 1434 |
| S15 | S3 AND S14 | 246 |

Table S4. Search strategy on ISI Web of Science.

Date searched: 19/12/25; 2207 results

| # | Search Query | Results |
| --- | --- | --- |
| 1 | TS=(osteoarthritis) | 254798 |
| 2 | TS=trapeziectom* | 968 |
| 3 | TS=(thumb or thumbs or trapezium or trapezia or trapeziums or carpometacarpal*) | 103812 |
| 4 | TS=(arthroplast* or prosthes* or prosthet* or replac* or surger* or procedur* or operation*) | 23364471 |
| 5 | #3 AND #4 | 27615 |
| 6 | #2 OR #5 | 27694 |
| 7 | #1 AND #6 | 2207 |

Table S5. Search strategy on Cochrane Central Register of Controlled Trials

Date searched: 19/12/25; 218 results

| ID | Search | Hits |
| --- | --- | --- |
| #1 | MeSH descriptor: [Osteoarthritis] this term only | 5597 |
| #2 | osteoarthritis:ti,ab,kw | 27313 |
| #3 | #1 or #2 | 27313 |
| #4 | MeSH descriptor: [Thumb] this term only | 265 |
| #5 | MeSH descriptor: [Trapezium Bone] this term only | 39 |
| #6 | MeSH descriptor: [Carpometacarpal Joints] this term only | 90 |
| #7 | (thumb or thumbs or trapezium or trapezia or trapeziums or carpometacarpal*):ti,ab,kw | 2735 |
| #8 | #4 or #5 or #6 or #7 | 2735 |
| #9 | MeSH descriptor: [Arthroplasty, Replacement] this term only | 233 |
| #10 | MeSH descriptor: [Joint Prosthesis] explode all trees | 2494 |
| #11 | (arthroplast* or prosthes* or prosthet* or replac* or surger* or procedur* or operation*):ti,ab,kw | 632909 |
| #12 | #9 or #10 or #11 | 632909 |
| #13 | #8 and #12 | 1172 |
| #14 | trapeziectom*:ti,ab,kw | 97 |
| #15 | MeSH descriptor: [Arthroplasty, Replacement, Finger] this term only | 13 |
| #16 | #14 or #15 | 109 |
| #17 | #13 or #16 | 1208 |
| #18 | #17 and #3 | 218 |

Table S6. Summary of characteristics of included studies

| Study Author | Study Type | Interventions | Type of Trapziectomy* | Prosthesis used | Immobilisation protocol | Patient number | Male:  Female patients | Age (mean) | Outcome measures** | Post-operative time points measured (months) | Key findings |
| --- | --- | --- | --- | --- | --- | --- | --- | --- | --- | --- | --- |
| Arasegawa 2023 | Single centre prospective cohort | Trapeziectomy | Modified Burton suspension arthroplasty | NA | 1-2 weeks | 26 | 7:19 | 67.3 | VAS, qDASH | 1, 3 | Improvement of pain and qDASH at 1 and 3 months |
| Assiotis 2017 | Single centre prospective cohort | Trapeziectomy | TightRope (Anthrex, Naples, FL) | NA | 1-2 weeks | 21 | 6:15 | 65.7 | Grip strength, key pinch strength | 3, 6, 12 | Improvemnet of grip strength and pinch strength by 12 months |
| Bonhof-Jansen 2024 | Single centre prospective cohort | Joint replacement (Supervised rehabilitation group) | NA | Uncemented Semi-constrained single mobility prosthesis (MAIA, Groupe Lepine, Genay, France) | 2-4 weeks | 31 | 0:31 | 58.6 | Grip strength, key pinch strength | 3, 12 | Improvement of grip strength and pinch strength by 12 months. There was no significant differences between groups. |
| Bonhof-Jansen 2024 | Single centre prospective cohort | Joint replacement (education alone group) | NA | Uncemented Semi-constrained single mobility prosthesis (MAIA, Groupe Lepine, Genay, France) | 2-4 weeks | 31 | 0:31 | 51.0 | Grip strength, key pinch strength | 3, 12 |  |
| Chang 2008 | Single centre prospective cohort | Trapeziectomy | Modified APL suspension arthroplasty | NA | NR | 18 | 4:14 | 59 | Grip strength, key pinch strength | 3, 6, 12 | Improvement of grip strength by 3 months |
| Colegate-Stone 2011 | Single centre prospective comparative cohort | Trapeziectomy | Simple trapeziectomy | NA | 2-4 weeks | 14 | 2:12 | 63.5 | Grip strength, key pinch strength | 6,12 | Both groups showed improvement in DASH, pain and strength measurements at 6 and 12 months. There were no significant differences between groups at each time-point. |
| Colegate-Stone 2011 | Single centre prospective comparative cohort | Trapeziectomy | Pyrocarbon interposition (medium size implant, P12, Tornier, San Diego, CA, USA; 9x13mm; large size 9x15mm) | NA | 2-4 weeks | 24 | 2:22 | 62 | Grip strength, key pinch strength | 6,12 |  |
| Davis 1997 | Single centre randomised prospective comparative cohort | Trapeziectomy | Simple trapeziectomy | NA | 2-4 weeks | 30 | 0:30 | 56.4 | Grip strength, key pinch strength | 3, 12 | All groups showed improvement in pain, hand function and strength measurements at 3 and 12 months. There were no significant differences between groups at each time-point. |
| Davis 1997 | Single centre randomised prospective comparative cohort | Trapeziectomy | With soft tissue interposition (palmaris longus tendon rolled up and sutured to palmar capsule) | NA | 2-4 weeks | 23 | 0:23 | 61.2 | Grip strength, key pinch strength | 3, 12 |  |
| Davis 1997 | Single centre randomised prospective comparative cohort | Trapeziectomy | With LRTI (Burton and Pelegrini technique) | NA | 2-4 weeks | 23 | 0:23 | 57.7 | Grip strength, key pinch strength | 3, 12 |  |
| Davis 2004 | Single centre randomised prospective comparative cohort | Trapeziectomy | Simple trapeziectomy | NA | 2-4 weeks | 62 thumbs | - | 58 | Grip strength, key pinch strength | 3, 12 | Key pinch strength improved in all groups by 12 months. There was no significant differences between groups. |
| Davis 2004 | Single centre randomised prospective comparative cohort | Trapeziectomy | With PL interposition | NA | 2-4 weeks | 59 thumbs | - | 60 | Grip strength, key pinch strength | 3, 12 |  |
| Davis 2004 | Single centre randomised prospective comparative cohort | Trapeziectomy | With LRTI with half the width of FCR | NA | 2-4 weeks | 62 thumbs | - | 59 | Grip strength, key pinch strength | 3, 12 |  |
|  |  | Trapeziectomy |  |  |  | Total: 162 patients | - |  |  |  |  |
| Davis and Pace 2009 | Single centre randomised prospective comparative cohort | Trapeziectomy | Simple trapeziectomy | NA | 2-4 weeks | 46 | 10:36 | 60 | DASH, grip strength, key pinch strength | 3, 12 | Both groups showed improvement in pain, DASH and strength measurements at 3 and 12 months. There were no significant differences between groups at each time-point. |
| Davis and Pace 2009 | Single centre randomised prospective comparative cohort | Trapeziectomy | With LRTI | NA | 2-4 weeks | 44 | 10:34 | 61 | DASH, grip strength, key pinch strength | 3, 12 |  |
| De Jong 2023 | Single centre double-blinded RCT | Trapeziectomy | Simple trapeziectomy | NA | 2-4 weeks | 31 | 0:31 | 61 | DASH, grip strength, key pinch strength | 3, 12 | Both groups showed improvement in DASH and strength measurements at 3 and 12 months. The joint replacement arm showed significantly more improvement than the trapeziectomy arm at each time-point. |
| De Jong 2023 | Single centre double-blinded RCT | Joint replacement | NA | Maia prosthesis (Groupe Lépine, Genay, France); uncemented; semi-constrained single mobility | 2-4 weeks | 31 | 0:31 | 59 | DASH, grip strength, key pinch strength | 3, 12 |  |
| Dremstrup 2021 | Single centre prospective cohort | Joint replacement | NA | Moovis cup; **uncemented**; semi-constrained dual mobility | 2-4 weeks | 168 | 37: 131 | 59 | Grip strength, qDASH | 3, 12 | Significant improvement of qDASH at 3 months, and grip strength at 12 months. Outcomes continued to improve to 24 months. |
| Esteban-Feliu 2021 | Single centre prospective randomised comparative cohort | Trapeziectomy | With LRTI, single-incision carpal tunnel release basal joint arthroplasty | NA | 1-2 weeks | 20 | 4:16 | 61.5 | Boston Carpal Tunnel Questionnaire, qDASH, 10-point VAS*** | 3, 6, 12 | Both groups showed improvement in BCTQ, qDASH and pain at 3, 6 and 12 months, with no statistical differences between groups at each time-point. |
| Esteban-Feliu 2021 | Single centre prospective randomised comparative cohort | Trapeziectomy | With LRTI, double-incision carpal tunnel release basal joint arthroplasty | NA | 1-2 weeks | 20 | 3:17 | 58.5 | Boston Carpal Tunnel Questionnaire, qDASH, 10-point VAS | 3, 6, 12 |  |
| Falkner 2024 | Non-randomised  2-centre prospective | Trapeziectomy | Simple (resection) arthroplasty | NA | >4 weeks | 22 | 3: 19 | 56 | Grip strength | 1.5, 3, 6, 12 | Improvment of grip strength above baseline strength at 3 months for prosthesis group, with resection arthroplasty group not recovering to baseline until 36 months. |
| Falkner 2024 | Non-randomised  2-centre prospective | Joint replacement | NA | TOUCH dual mobility prosthesis, KeriMedical, Switzerland | >4 weeks | 47 | 12: 35 | 58 | Grip strength | 1.5, 3, 6, 12 |  |
| Frost 2023 | Single centre prospective non-randomised cohort | Trapeziectomy | With LRTI (Burton and Pelegrini technique) | NA | >4 weeks | 20 | 8:12 | 64 | Grip strength, key pinch strength | 3, 6, 12 | Key pinch showed steady improvement at 3, 6 and 12 months. Grip strength showed improvement from baseline to 3 months but returned to baseline by 6 months. Improvement was noted at 12 and 24 months. |
| Frouzakis 2015 | Single centre prospective comparative cohort | Trapeziectomy | With LRTI | NA | NR | 105 | 13: 92 | 64 | Michigan Hand Outcomes Questionnaire (MHQ), key pinch strength | 3, 6, 12 | Improvement of MHQ scores at 3, 6 and 12 months. |
| Fulchignomi 2021 | Single centre prospective cohort | Trapeziectomy | Suspension arthroplasty (Brunelli technique: APL palmar slip suspension ligamen) | NA | 2-4 weeks | 67 | 17: 50 | 64 | Grip strength, key pinch strength | 12 | Improvement of strength measurements at 12 months. |
| Graesser 2024 | RCT | Trapeziectomy | With LRTI | NA | 2-4 weeks | 15 | 3:12 | 61 | Grip strength, key pinch strength | 1, 3, 12 | Grip strength and key pinch strength showed deterioration from baseline at 1 month, but returned to baseline strength at 3 months. |
| Guzzini 2023 | Single centre prospective comparative cohort | Trapeziectomy | With APL interposition arthroplasty | NA | 2-4 weeks | 71 | 13:58 | 66 | DASH, grip strength, key pinch strength | 1, 3, 6, 12 | Both groups showed improvement in strength measurements and range of motion at 12 months, with the joint replacement group showing significantly more improvement compared to trapeziectomy. |
| Guzzini 2023 | Single centre prospective comparative cohort | Joint replacement | NA | Touch TMC joint press-fit conical cups (KeriMedical; Geneva, Switzerland); dual mobility | <1 week | 65 | 14:54 | 68 | DASH, grip strength, key pinch strength | 1, 3, 6, 12 |  |
| Hansen and Stilling 2013 | Single centre prospective parallel group patient blinded RCT | Joint replacement | NA | Elektra screw cup (Small Bone Innovations Inc., Les Bruyeres, France); cemented DLC all-polyethylene cup | 2-4 weeks | 14 | 2:12 | 56 | DASH, grip strength | 3, 6, 12 | Both groups showed improvement in DASH at 3, 6 and 12 months. Both groups showed deterioration in grip strength at 3 months, followed by improvement beyond baseline at 6, and continued improvement to 12 months. |
| Hansen and Stilling 2013 | Single centre prospective parallel group patient blinded RCT | Joint replacement | NA | Elektra screw cup (Small Bone Innovations Inc., Les Bruyeres, France); uncemented Elektra chrome-cobalt grit-blasted hydroxy- apatite-coated screw cup | 2-4 weeks | 13 | 1:12 | 60 | DASH, grip strength | 3, 6, 12 |  |
| Hansen and Kirkeby 2016 | Single centre prospective comparatice cohort | Joint replacement (Easton-Glickel stage 2) | NA | Elektra press-fit stem uncemented screwed cup or cemented DLC cup (Small Bone Innovations Inc., Les Bruyeres, France); Motec uncemented screwed step and cup (Swemac Orthopaedics AB, Linkoping, Sweden) | 2-4 weeks | 26 | 3:23 | 55 | DASH | 3, 6, 12 | All groups showed improvement in DASH score at 3, 6 and 12 months. For grip strengths, all groups showed deterioration at 3 months. All groups showed improvement from baseline at 12 months. No significant differences in DASH and grip strength between groups at 12 months. |
| Hansen and Kirkeby 2016 | Single centre prospective comparatice cohort | Joint replacement (Easton-Glickel stage 3) | NA | Elektra press-fit stem uncemented screwed cup or cemented DLC cup (Small Bone Innovations Inc., Les Bruyeres, France); Motec uncemented screwed step and cup (Swemac Orthopaedics AB, Linkoping, Sweden) | 2-4 weeks | 30 | 5:25 | 60 | DASH | 3, 6, 12 |  |
| Hansen and Kirkeby 2016 | Single centre prospective comparatice cohort | Joint replacement (Easton-Glickel stage 4) | NA | Elektra press-fit stem uncemented screwed cup or cemented DLC cup (Small Bone Innovations Inc., Les Bruyeres, France); Motec uncemented screwed step and cup (Swemac Orthopaedics AB, Linkoping, Sweden) | 2-4 weeks | 13 | 6: 7 | 66 | DASH | 3, 6, 12 |  |
| Hermann-Eriksen 2022 | Single centre prospective non-inferiority RCT | Trapeziectomy | Trapeziectomy (control group) | NA | 1-2 weeks | 29 | 4:25 | 62.1 | Grip strength | 3, 6, 12 | All groups showed improvement in grip strength at 12 months. No significant differences between groups at 12 months. |
| Hermann-Eriksen 2022 | Single centre prospective non-inferiority RCT | Trapeziectomy | Trapeziectomy (intervention group) | NA | 1-2 weeks | 29 | 4:25 | 66.7 | Grip strength | 3, 6, 12 |  |
| Jaatinen 2025 | Muti-centre prospective cohort | Trapeziectomy | Simple trapeziectomy | NA | >4 weeks | 52 | 13: 39 | 62 | PRWHE pain and function scale, NRS pain and function scale | Every month for the first 12 months. | No improvement of pain and hand function in first 6 weeks with use of orthosis. |
| Janakiramanan 2021 | Single centre prospective cohort | Trapeziectomy | With LRTI | NA | 1-2 weeks | 55 | 10:45 | 63 | DASH, grip strength, key pinch strength | 3, 6, 9, 12 | Significant improvement of DASH from 3-9 months. Grip strength exceeded pre-operative strength at 6 months, with no change in key pinch strength. |
| Joosten 2025 | Single centre prospective cohort | Joint Replacement | NA | CarpoFit (Implantcast, Buxtehude, Germany) | NR | 292 | 36:256 | 66.4 | Grip strength | 3, 6, 12 | Improvement of grip strength at 3 months. |
| Jorgensen 2021 | Single centre prospective cohort | Trapeziectomy | With FCR tendon interposition (Burton and Pellegrini technique) | NA | NR | 180 | NR | NR | 5-point pain scale, qDASH | 6,12 | All groups showed improvement of pain and qDASH at 6 and 12 months. No significant differences in pain and qDASH between groups at each time-point. |
| Jorgensen 2021 | Single centre prospective cohort | Trapeziectomy | Operated with Weibly technique | NA | NR | 110 | NR | NR | 5-point pain scale, qDASH | 6,12 |  |
| Jorgensen 2021 | Single centre prospective cohort | Trapeziectomy | With capsuloplasty | NA | NR | 67 | NR | NR | 5-point pain scale, qDASH | 6,12 |  |
|  |  | Trapeziectomy |  |  |  | 357 | 82: 257 | 62.5 | 5-point pain scale, qDASH | 6,12 |  |
| Jorgensen 2024 | Single centre prospective comparative cohort | Trapeziectomy | With LRTI 2 button and Mini TightRope system (Arthrex) | NA | NA | 12 | 4: 8 | 53.5 | 5-point pain scale, qDASH | 1.5,3,6,12 | Both groups showed improvement of qDASH score at 12 months. There was no significant difference in qDASH scores between groups at 12 months. |
| Jorgensen 2024 | Single centre prospective comparative cohort | Trapeziectomy | With LRTI | NA | >4 weeks | 36 | 12:24 | 57.5 | 5-point pain scale, qDASH | 1.5,3,6,12 |  |
| Klim 2023 | Single centre RCT | Trapeziectomy | Epping resection-suspension arthroplasty | NA | NA | 86 | 19: 67 | 60 | DASH | 3, 6, 12 | Both groups showed improvement in DASH at 3, 6 and 12 months, with significantly more improvement in DASH for the prosthesis group compared to Epping arthroplasty group at 6 weeks. There were no significant differences between groups at 6 and 12 months. |
| Klim 2023 | Single centre RCT | Joint replacement | NA | Moovis cup, dual mobility (Stryker, Kalamazoo, MI, USA) | NA | 82 | 13: 69 | 56 | DASH | 3, 6, 12 |  |
| Komura 2020 | Prospective cohort | Trapeziectomy | With LRTI | NA | 2-4 weeks | 19 | 2: 17 | 67.3 | DASH, grip strength, key pinch strength | 3, 6, 12 | Grip and pinch strength showed deterioration at 3 months, improvement to baseline at 6 months and significant improvement at 12 months. DASH showed significantly more improvement at 3 months and continued to improve to 12 months. |
| Komura 2023 | Single centre prospective cohort | Trapeziectomy | With LRTI | NA | 2-4 weeks | 16 | 2: 14 | 64.9 | DASH, grip strength, key pinch strength | 12 | DASH and strength measurements showed improvement at 12 months and continued to improve to 2 and 3 years |
| Komura 2024 | Single centre prospective cohort | Trapeziectomy | With LRTI | NA | 2-4 weeks | 11 | 0: 11 | 62.5 | DASH, grip strength, key pinch strength | 12 | Improvement of DASH, grip strength and key pinch strength by 12 months. |
| Krasny 2025 | Single centre prospective cohort | Trapeziectomy | With interposition arthroplasty with no orthotic thumb device | NA | 2-4 weeks | 15 | 4:11 | 61.5 | Grip strength |  | All groups showed deterioration in grip strength from baseline at 6 weeks, followed by improvement in strength at 12 weeks, with only the short orthotic thumb device group returning to baseline strength. |
| Krasny 2025 | Single centre prospective cohort | Trapeziectomy | With interposition arthroplasty with short orthotic thumb device | NA | 2-4 weeks | 15 | 3:12 | 58.3 | Grip strength |  |  |
| Krasny 2025 | Single centre prospective cohort | Trapeziectomy | With interposition arthroplasty with long orthotic thumb device | NA | 2-4 weeks | 12 | 2:10 | 64.1 | Grip strength |  |  |
| Larson 2022 | Single centre level I inception cohort | Trapeziectomy | Simple trapeziectomy(high STarT Psych-sub groups) | NA | NR | 24 | 3: 21 | 65.5 | Patient Evaluation Measure (PEM), 10-point pain scale, qDASH | 1.5, 4. 12 | Both groups showed improvement in pain and qDASH scores at 4 and 12 months. |
| Larson 2022 | Single centre level I inception cohort | Trapeziectomy | Simple trapeziectomy(low STarT Psych-sub groups) | NA | NR | 59 | 9: 50 | 66.5 | Patient Evaluation Measure (PEM), 10-point pain scale, qDASH | 1.5, 4. 12 |  |
| Li 2019 | Prospective cohort | Trapeziectomy | With LRTI | NA | >4 weeks | 17 | NR: NR | NR | DASH, grip strength | 12 | Improvement in DASH and strength measurements at 12 months. |
| Marks 2017 | Single centre RCT | Trapeziectomy | With FCR tedon (modified Weilby technique) | NA | NA | 29 | 3: 26 | 64 | DASH, grip strength, key pinch strength | 1.5, 3, 6, 12 | For DASH, both groups showed deterioration in scores at 6 weeks, followed by improvement at 3 and 6 months. For strength measurements, there was a deterioration from baseline at 3 months, followed by improvement at 6 and 12 months. |
| Marks 2017 | Single centre RCT | Trapeziectomy | GraftJacket allograft for suspension interposition (Wright Medical Group, Inc., Memphis, TN)(modified Weilby technique) | NA | NA | 31 | 6: 25 | 68 | DASH, grip strength, key pinch strength | 1.5, 3, 6, 12 |  |
| McCullough 2021 | Single centre, non-randomised controlled trial | Trapeziectomy | Simple trapeziectomy | NA | >4 weeks | 7 | 2: 5 | 65.7 | DASH, grip strength, key pinch strength | 12 | For DASH, both groups showed slight deterioration at 6 weeks, followed by significant improvement at 6 months. At 12 months, there was a plateau in the meniscus group and slight improvement for the control group. Strength deteriorated to 6 weeks for both groups, followed by significant improvement at 6 months, followed by a plateau. |
| McCullough 2021 | Single centre, non-randomised controlled trial | Trapeziectomy | With interposition arthroplasty with meniscal allograft | NA | >4 weeks | 23 | 6: 17 | 61.4 | DASH, grip strength, key pinch strength | 12 |  |
| Miura 2008 | Single centre prospective cohort | Trapeziectomy | With LRTI | NA | 2-4 weeks | 10 | 0: 10 | 61 | Grip strength, key pinch strength | 2, 4, 6, 12 | Grip strength and key pinch strength deteriorated slightly at 2 months, followed by improvement at 4, 6 and 12 months. |
| Nilsson 2010 | Multi-centre, observer-blinded RCT | Trapeziectomy | With tendon interposition arthroplasty (APL, ECRL and Burton procedure) | NA | NR | 37 | 4: 33 | 61 | Grip strength, key pinch strength | 12 | Improvement of pain at 3, 6, 9 and 12 months. Strength showed deterioration at 3 months, followed by improvement to baseline at 6 months and significant improvement at 12 months. |
| Piccirilli 2024 | Two-centre prospective cohort | Joint Replacement | NA | Touch (KeriMedical) prosthesis | NR | 50 | 8:42 | 59.2 | VAS, qDASH, Kapandji | 1, 3, 6, 12 | The joint replacement group showed significant improvment of qDASH score at 1 and 6 months compared to trapeziectomy group. |
| Piccirilli 2024 | Two-centre prospective cohort | Trapeziectomy | With tendon interposition arthroplasty | NA | NR | 50 | 10:40 | 63.3 | VAS, qDASH, Kapandji | 1, 3, 6, 12 |  |
| Picchi 2025 | RCT | Trapeziectmy | With suspension tenoplasty using FCR tension (Altissimi technique) | NA | 2-4 weeks | 18 | 4:14 | 60.3 | DASH, grip strength | 3, 6, 12 | Both groups showed improvement in DASH score at 3 months, with no significant difference between groups. |
| Picchi 2025 | RCT | Joint Replacement | NA | MAIA dual mobility prosthesis (Group Lépin, Genay Cedex, France) | 2-4 weeks | 18 | 6:12 | 61.2 | DASH, grip strength | 3, 6, 12 |  |
| Reischenbock 2024 | Single centre prospective | Joint replacement | NA | Touch prosthesis (KeriMedical, Geneva, Switzerland), dual mobility | <1 week | 131 | 37: 94 | 63 | Key pinch strength | 1.5, 12 | Both groups showed improvement at 6 weeks and 12 months, with capsular resection group showing significantly more improvement at 6 weeks compared to capsular suturing group. |
| Reischenbock 2024 | Single centre prospective | Joint replacement | NA | Touch prosthesis (KeriMedical, Geneva, Switzerland), dual mobility | <1 week | 57 | 14: 43 | 61 | Key pinch strength | 1.5, 12 |  |
| Reissner 2015 | Single centre prospective comparative cohort | Trapeziectomy | With LRTI | NA | NR | 105 | 92: 13 | 64 | Key pinch strength | 12 | Improvement of key pinch strength at 12 months. |
| Rocchi 2011 | Single centre prospective cohort | Trapeziectomy | Looping of APL tendon around 1st intermetacarpal ligament without requiring bone tunneling/looping around a tendon | NA | <1 week | 50 | 8: 34 | 60 | DASH, grip strength, key pinch strength | 3, 6, 12 | Improvement of DASH at 3, 6 and 12 months. Improvement of strength measurements at 12 months. |
| Sanchez-Flo 2020 | Single centre, single blinded RCT | Trapeziectomy | With LRTI (suspension and interposition arthroplasty) (Weilby technique) | NA | 2-4 weeks | 17 | 2: 15 | 61 | Grip strength, key pinch strength | 12 | Deterioration of key pinch strength from baseline to 12 months. |
| Sirotakova et al 2007 | Single centre prospective cohort | Trapeziectomy | With APL sling arthroplasty | NA | 2-4 weels | 74 | 15: 59 | 60 | Grip strength, key pinch strength | 6, 12 | Deterioration of grip and key pinch strength at 6 months, followed by improvement to 12 months. |
| Shonuga 2021 | Single centre prospective comparative cohort | Trapeziectomy | APL group, suspensionplasty as described by Thompson and Brunelli | NA | NR | 34 | NR | 63.8 | VAS, qDASH, key pinch strength | 3, 12 | Both groups showed deterioration in qDASH at 1 month, followed by improvement at 3 and 12 months, with the tightrope group showing more improvement compared to the other groups at 2 weeks, 3 months and 12 months. |
| Shonuga 2021 | Single centre prospective comparative cohort | Trapeziectomy | FCR group, LRTI as described by Burton and Pellegrini | NA | NR | 19 | NR | 64.9 | VAS, qDASH, key pinch strength | 3, 12 |  |
| Shonuga 2021 | Single centre prospective comparative cohort | Trapeziectomy | TightRope group | NA | NR | 59 | NR | 62.2 | VAS, qDASH, key pinch strength | 3, 12 |  |
|  |  | Trapeziectomy |  |  |  |  | 32: 80 |  |  |  |  |
| Stirton 2022 | Single centre, prospective randomised cohort | Trapeziectomy | With LRTI (accelerated group) | NA | 2-4 weeks | 48 thumbs | NR | 60.1 | DASH, grip strength, key pinch strength | 1.5, 3, 6, 12 | The accelerated group showed most improvement in DASH and grip strength compared to traditional group at 6 weeks, but the scores were equivalent by 3 months. |
| Stirton 2022 | Single centre, prospective randomised cohort | Trapeziectomy | With LRTI (traditional rehabilitation group) | NA | >4 weeks | 45 thumbs | NR | 58.9 |  |  |  |
|  |  | Trapeziectomy |  |  |  | 90 | 17: 73 | NR |  |  |  |
| Suszynski 2023 | Single centre prospective comparative cohort | Trapeziectomy | With LRTI | NA | 2-4 weeks | 26 | 8: 18 | 63 | DASH, grip strength | 1.5, 6 | Both groups showed improvement of DASH at 6 weeks and 6 months. Both groups showed a deterioration of grip strength at 6 weeks followed by improvement above baseline by 6 months. |
| Suszynski 2023 | Single centre prospective comparative cohort | Trapeziectomy | With suture-only suspension arthroplasty | NA | 2-4 weeks | 19 | 5: 14 | 61.5 | DASH, grip strength | 1.5, 6 |  |
| Tagil and Kopylov 2002 | Single centre prospective randomised cohort | Trapeziectomy | APL tendon interposition arthroplasty | NA | NR | 13 | 1: 12 | NR | Grip strength | 6 | Improvement in grip strength at 6 months. No significant differences between groups at 6 months. |
| Tagil and Kopylov 2002 | Single centre prospective randomised cohort | Joint replacement | NA | Swanson silicone trapezium implant | NR | 13 | 1: 12 | NR | Grip strength | 6 |  |
|  |  |  |  |  |  |  | - | 62 |  |  |  |
| Ten Binke 2016 | Single centre prospective cohort | Joint replacement | NA | SRTMC, Avanta TMC joint prosthesis | NR | 10 | 1: 9 | NR | DASH | 1.5, 6, 12 | Improvement in DASH at 6 and 12 months. |
| Thorkildsen 2019 | Single centre RCT | Joint replacement | NA | Hydroxyapatite-coated Elektrra (Les Bruyeres, France); uncemented | >4 weeks | 20 | 6: 14 | 64 | Grip strength, key pinch strength | 3, 6, 12 | Both groups showed deterioration of grip strength at 3 months, followed by improvement at 6 months. At 12 months, grip strength continued to improve for trapeziectomy but showed slight deterioration for joint replacement. There were no significant differences between grops at each time-point. Slight improvement in key pinch strength for joint replacement group at 3, 6 and 12 months, compared to slight deterioration for trapeziectomy at 3, 6 and 12 months. |
| Thorkildsen 2019 | Single centre RCT | Trapeziectomy | With LRTI | NA | NR | 20 | 6: 14 | 61 | Grip strength, key pinch strength | 3, 6, 12 |  |
| Tsehaie 2019 | Multi-centre prospetive cohort | Trapeziectomy | With tendon interposition arthroplasty (Weilby technique) | NA | 2-4 weeks | 233 | 42: 191 | 59 | Key pinch strength | 12 | Improvement of key pinch strength at 12 months. |
| Ulrich-Vinter 2008 | Single centre prospective cohort | Trapeziectomy | With tendon interposition arthroplasty | NA | 2-4 weeks | 70 | 11: 59 | 62 | Grip strength, key pinch strength | 3, 6, 12 | For key pinch strength, the joint replacement group showed improvement at 3, 6 and 12 months. The arthroplasty group showed deterioration at each time-point without return to baseline. For grip strength, the joint replacement group showed improvement at 3, 6 and 12 months, whereas the trapeziectomy group showed deterioration at 3 months followed by improvement at 6 and 12 months. |
| Ulrich-Vinter 2008 | Single centre prospective cohort | Joint replacement | NA | Elektra, uncemented, press fit technique | 2-4 weeks | 42 | 5: 37 | 58 | Grip strength, key pinch strength | 3, 6, 12 |  |
| Vadstrup 2009 | Single centre prospective cohort | Trapeziectomy | With tendon interposition arthroplasty (Weilby technique) | NA | 2-4 weeks | 105 | 18: 87 | 61 | Grip strength | 1.5, 3, 6,12 | Significant improvement in pain at 3 motnhs. Grip strength reached baseline values at 4-6 months and conitnued to improve to 12 months. |
| Van Laarhoven 2023 | Multi-centre RCT | Trapeziectomy | Open trapeziectomy | NA | 2-4 weeks | 42 | 7: 35 | 62 | Grip strength, key pinch strength | 3, 12 | Deterioration of strength measurements at 3 months followed by improvement at 12 months. |
| Van Royen 2018 | Single centre prospective cohort | Trapeziectomy | With poly-L/D-lactide spacer | NA | 2-4 weeks | 9 | 0: 9 | 64 | Grip strength, key pinch strength | 6, 12 | Grip strength deteriorated at 6 months, followed by slight improvement below baseline at 12 months. Key pinch strength showed deterioration from baseline at 6 and 12 months. |
| Vermeulen 2009 | Single centre prospectice single-arm cohort study | Trapeziectomy | With Weilby interposition tendoplasty | NA | 2-4 weeks | 19 | 2: 17 | 58 | DASH, grip strength, key pinch strength | 3, 6, 12 | Improvement of DASH and key pinch strength at 3, 6 and 12 months. Grip strength showed deterioration at 3 months followed by improvement at 6, 9 and 12 months. |
| Vermeulen 2014 | Single centre, single blinded, parallel group RCT | Trapeziectomy | With LRTI | NA | 2-4 weeks | 21 | 0: 21 | 59 | DASH, grip strength, key pinch strength | 3, 12 | Improvement of DASH at 3 and 12 months. Deterioration of strength measurements at 3 months followed by improvement by 12 months |
| Vermeulen 2014 | Single centre, single blinded, parallel group RCT | Trapeziectomy | With Burton-Pellegrini technique | NA | 2-4 weeks | 36 | 0: 36 | 64.7 | DASH, grip strength, key pinch strength | 3, 12 | Both groups showed improvement in DASH at 3 and 12 months. Both groups showed deterioration in strength measurements at 3 months, followed by improvement to 12 months. No significant differences in either outcome between groups at each time-point. |
| Vermeulen 2014 | Single centre, single blinded, parallel group | Trapeziectomy | With Weilby technique | NA | 2-4 weeks | 36 | 0: 36 | 63.5 | DASH, grip strength, key pinch strength | 3, 12 |  |
| Wilcke 2020 | Prospective registry-based study | Trapeziectomy | With LRTI | NA | NR | 1850 | 386: 1464 | 63 | Pain at rest, in motion wihtout load and with load, qDASH | 3, 12 | Improvement in pain and qDASH at 3 and 12 months. |
| Wilcke 2022 | Prospective registry-based study | Trapeziectomy | Simple trapeziectomy | NA | NR | 59 | 0: 59 | 66 | Grip strength, key pinch strength | 3, 12 | All groups showed improvement in pain and strength measurements at 3 and 12 months. No significant differences in strength measurements between groups at 3 and 12 months. |
| Wilcke 2022 | Prospective registry-based study | Trapeziectomy | With FCR tendon adjunct | NA | NR | 233 | 0: 233 | 64 | Grip strength, key pinch strength | 3, 12 |  |
| Wilcke 2022 | Prospective registry-based study | Trapeziectomy | With ECRL tendon adjunct | NA | NR | 183 | 0: 183 | 63 | Grip strength, key pinch strength | 3, 12 |  |
| Wilcke 2022 | Prospective registry-based study | Trapeziectomy | With APL tendon adjunct | NA | NR | 185 | 0: 185 | 63 | Grip strength, key pinch strength | 3, 12 |  |
| Zarezadeh 2021 | Single centre prospective clinical trial | Trapeziectomy | With hematoma distraction arthroplasty (suspension arthroplasty) | NA | 2-4 weeks | 28 | 8: 20 | 54.32 | DASH | 3, 6, 12 | Both groups showed improvement in DASH at 3, 6 and 12 months, with hematoma distraction arhtroplasty grop showing more improvement at 12 months. |
| Zarezadeh 2021 | Single centre prospective clinical trial | Trapeziectomy | With LRTI | NA | 2-4 weeks | 28 | 10: 18 | 54.64 | DASH | 3, 6, 12 |  |

*APL: abductor pollicis longus, LRTI: ligament reconstruction and tendon interposition, FCR: flexor carpi radialis, PL: palmaris longus, ECRL: extensor carpi radialis longus.

**The outcomes listed are those relevant to the review and not necessarily all the outcomes captured by the study; qDASH: quick DASH, VAS: Visual Analogue Scale.

Table S7. Risk of bias assessment of included studies.

| Study author | Study arm | ROB assessment* | Objective clearly stated | Pre-specified eligibility criteria | Represent-ative sample | All eligible participants enrolled | N>100 | Intervent-ion clearly described & consiste-ntly delivered | Consistent outcome measure-ment | Blinding | Attrition <20% |
| --- | --- | --- | --- | --- | --- | --- | --- | --- | --- | --- | --- |
| Chang et.al. 2008 | Trapeziectomy with modified APL suspension arthroplasty | High ROB | + | - | + | + | - | + | + | - | + |
| de Jong et al. 2023 | Trapeziectomy | Moderate ROB | + | + | + | + | - | + | + | + | + |
| de Jong et al. 2023 | Joint replacement | Moderate ROB | + | + | + | + | - | + | + | + | + |
| Esteban-Feliu et al. 2021 | Single incision carpal tunnel release | High ROB | + | + | + | + | - | + | + | - | + |
| Esteban-Feliu et al. 2021 | Double incision carpal tunnel release | High ROB | + | + | + | + | - | + | + | - | + |
| Fulchignoni et al. 2021 | APL suspension arthroplasty | High ROB | + | + | + | + | - | + | + | - | - |
| Guzzini et al. 2023 | Interposition arthroplasty | High ROB | + | + | + | + | - | + | + | - | + |
| Guzzini et al. 2023 | Dual cup mobility prosthesis | High ROB | + | + | + | + | - | + | + | - | + |
| Graesser et al. 2024 | Trapeziectomy + LRTI | High ROB | + | + | + | + | - | + | + | - | + |
| Hansen et al. 2016 | Eaton-Glickel stage 2 | High ROB | + | + | + | + | - | + | + | - | + |
| Hansen et al. 2016 | Eaton-Glickel stage 3 | High ROB | + | + | + | + | - | + | + | - | + |
| Hansen et al. 2016 | Eaton-Glickel stage 4 | High ROB | + | + | + | + | - | + | + | - | + |
| Komura et al. 2020 | Trapeziectomy + LRTI | High ROB | + | + | + | + | - | + | + | - | + |
| Komura et al. 2023 | Trapeziectomy + LRTI | High ROB | + | + | + | + | - | + | + | - | - |
| Komura et al. 2024 | Trapeziectomy + LRTI | High ROB | + | + | + | + | - | + | + | - | + |
| Li et al. 2019 | Joint replacement | High ROB | + | + | + | + | - | + | + | - | + |
| Marks et al. 2017 | Trapeziectomy + FCR tendon | High ROB | + | + | + | + | - | + | + | - | + |
| Marks et al. 2017 | Trapeziectomy + allograft for suspension interposition | High ROB | + | + | + | + | - | + | + | - | + |
| McCullough et al. 2023 | Trapziectomy | Moderate ROB | + | + | + | + | - | + | + | + | + |
| McCullough et al. 2023 | Trapeziectomy + interposition arthroplasty with meniscus | Moderate ROB | + | + | + | + | - | + | + | + | + |
| Nilsson et al. 2010 | Tendon interposition arthroplasty | Moderate ROB | + | + | + | + | - | + | + | + | + |
| Nilsson et al. 2010 | Artelon CMC spacer arthroplasty | Moderate ROB | + | + | + | + | - | + | + | + | + |
| Picchi et al. 2025 | Trapeziectomy | High ROB | + | + | + | + | - | + | + | - | + |
| Picchi et al. 2025 | Joint Replacement | High ROB | + | + | + | + | - | + | + | - | + |
| Rocchi et al. 2011 | Trapeziectomy with APL interposition | High ROB | + | + | + | + | - | + | + | - | + |
| Sanchez-Flo et al. 2020 | Total trapeziectomy | Moderate ROB | + | + | + | + | - | + | + | + | + |
| Ten Brinke et al. 2016 | Arthroplasty | High ROB | + | + | + | + | - | + | + | - | + |
| Tsehaie et al. 2019 | Trapeziectomy | Moderate ROB | + | + | + | + | + | + | + | - | + |
| Ulrich-Vinther et al. 2008 | Resection arthroplasty | Moderate ROB | + | + | + | + | - | + | + | + | + |
| Ulrich-Vinther et al. 2008 | Elektra prosthesis | Moderate ROB | + | + | + | + | - | + | + | + | + |
| Van Royen et al. 2018 | Trapeziectomy + spacer | High ROB | + | + | + | + | - | + | + | - | + |
| Vermeulen et al. 2009 | Weilby interposition tendoplasty | High ROB | + | + | + | + | - | + | + | - | + |
| Vermeulen et al. 2014 | Trapeziectomy + LRTI | Moderate ROB | + | + | + | + | - | + | + | + | + |
| Vermeulen et al. 2014 | Trapeziectomy (BP) | High ROB | + | + | + | + | - | + | + | - | + |
| Vermeulen et al. 2014 | Trapeziectomy (W) | High ROB | + | + | + | + | - | + | + | - | + |
| Zarezadeh et al. 2021 | Hematoma distraction arthroplasty | Moderate ROB | + | + | + | + | - | + | + | + | + |
| Zarezadeh et al. 2021 | Trapeziectomy + LRTI | Moderate ROB | + | + | + | + | - | + | + | + | + |

* Studies were assessed to have a low bias if all questions were answered yes; moderate if one question was answered no and high if two or more questions were answered no. Symbols “+” and “-“ represent yes and no respectively

Figure S1. Forest and funnel plot of DASH score for trapeziectomy at 1 month.


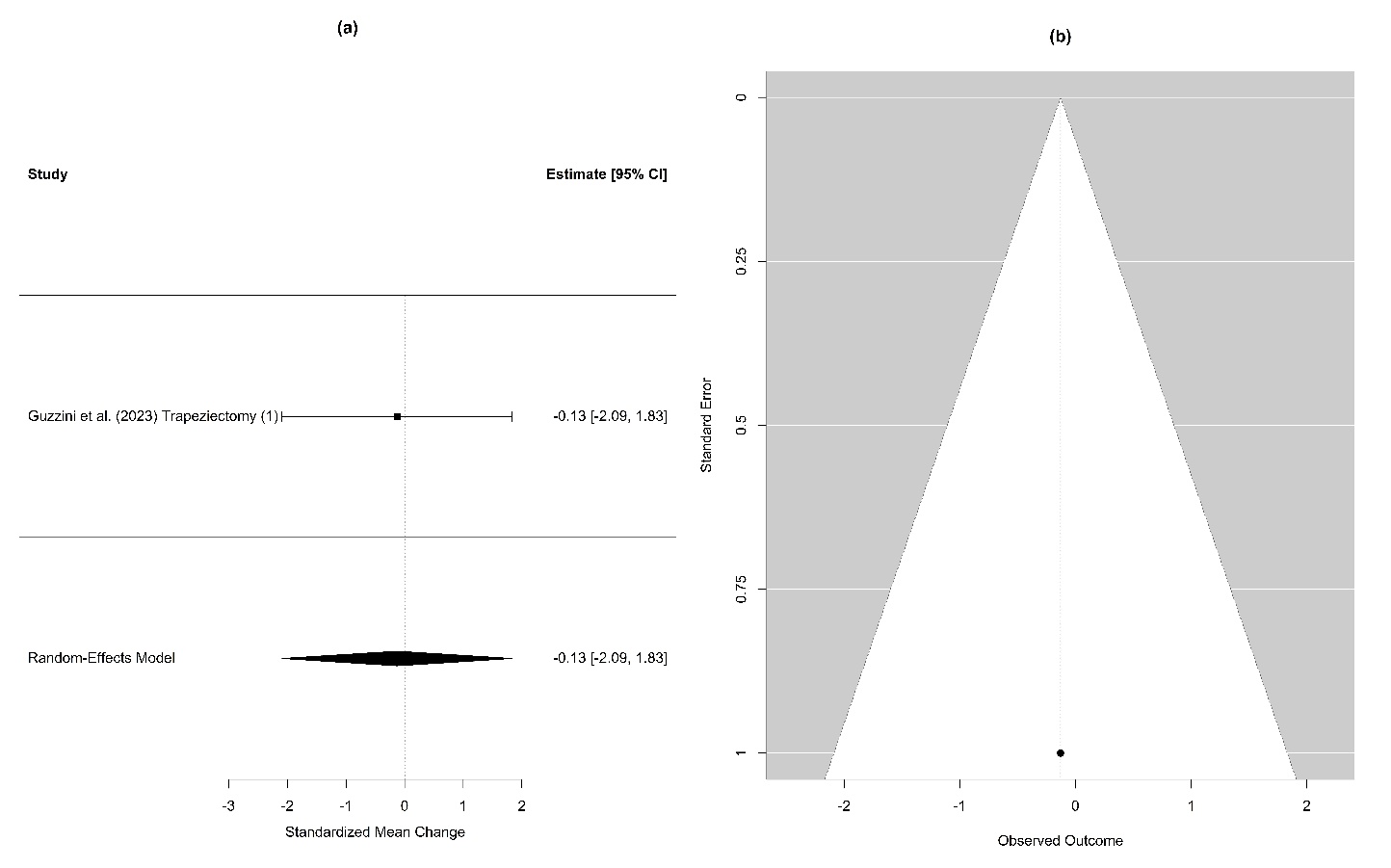


Figure S2. Forest and funnel plot of DASH score for trapeziectomy at 1.5 months.


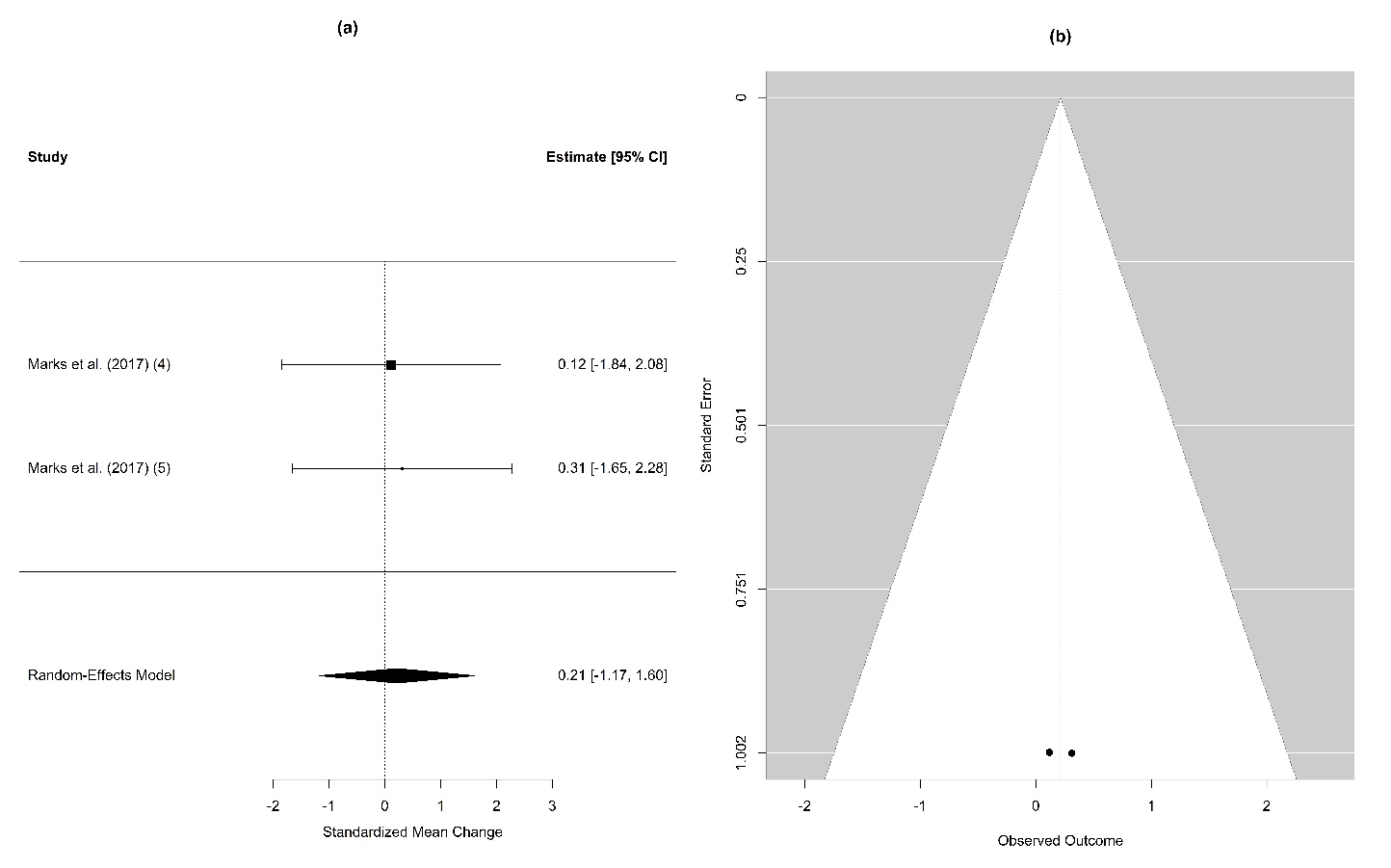


Figure S3. Forest and funnel plot of DASH score for trapeziectomy at 3 months.


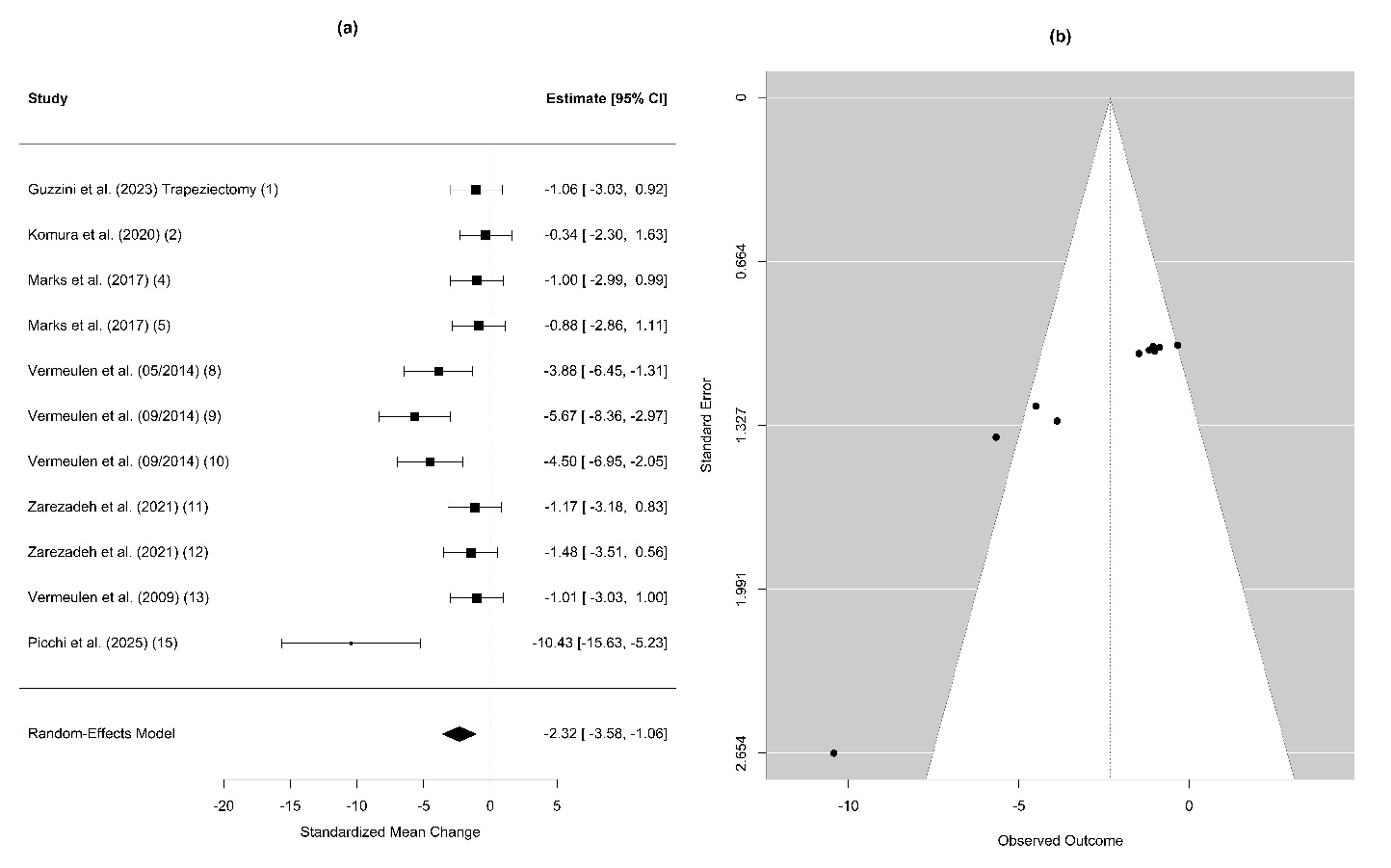


Figure S4. Forest and funnel plot of DASH score for trapeziectomy at 6 months.


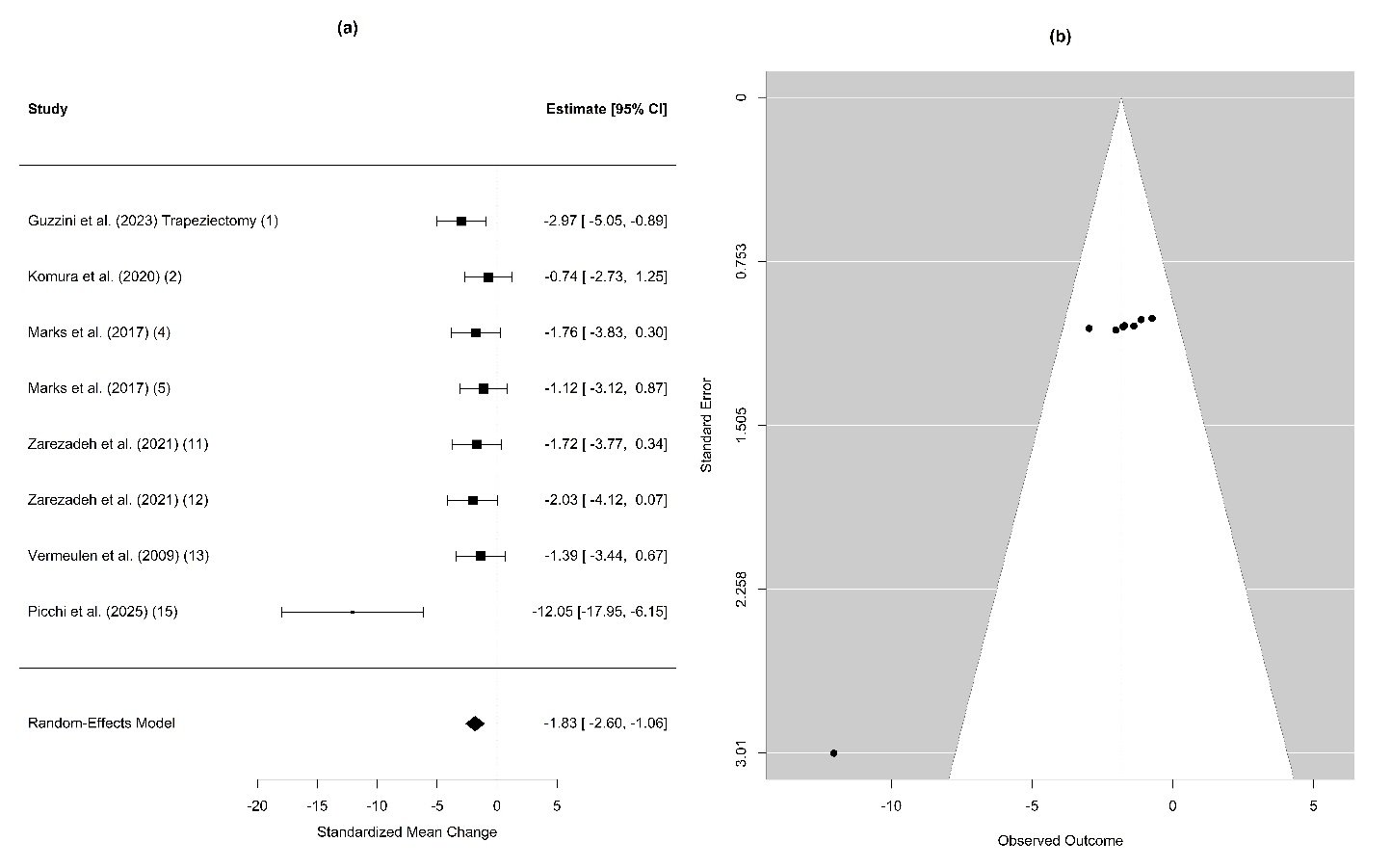


Figure S5. Forest and funnel plot of DASH score for trapeziectomy at 12 months.


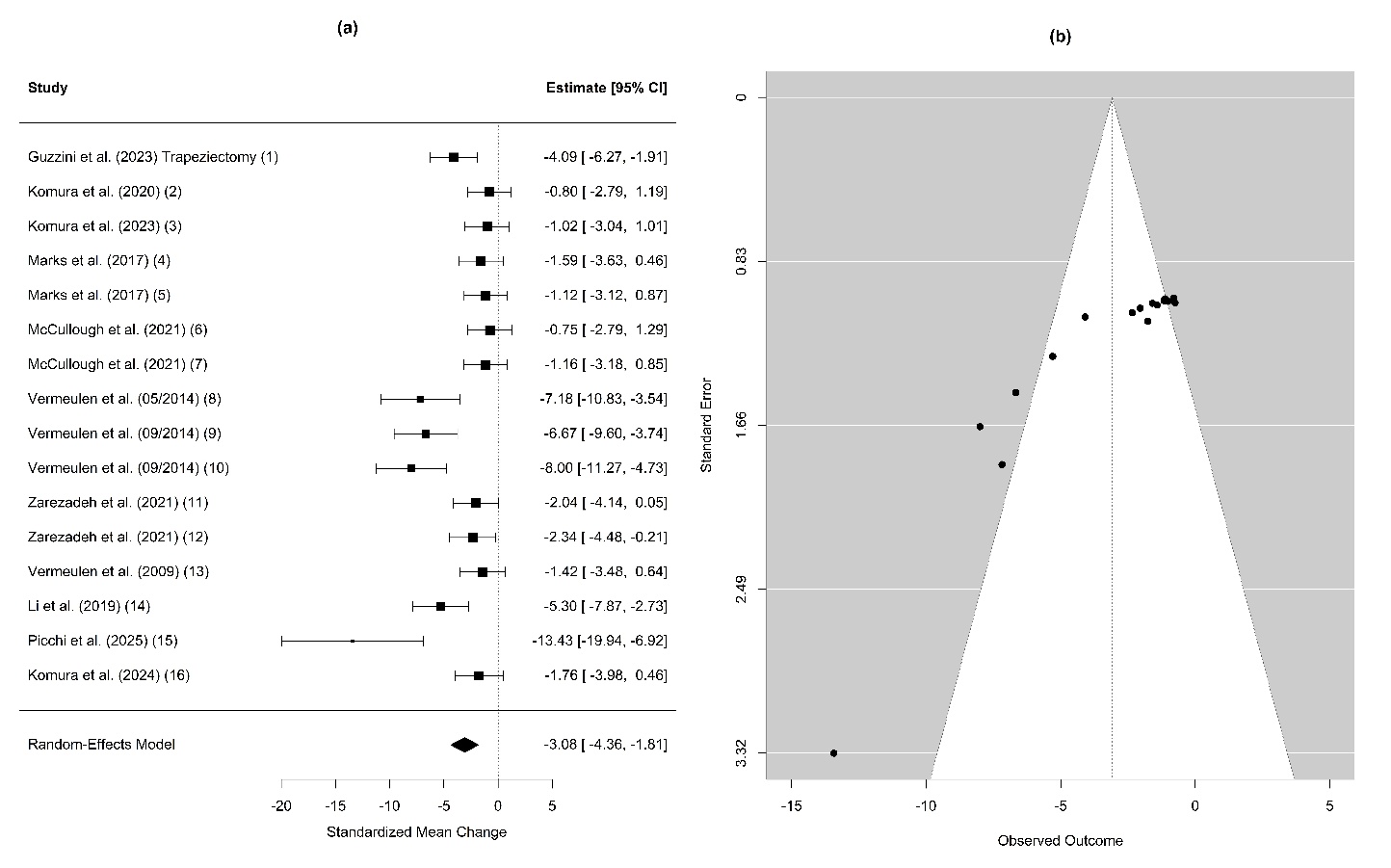


Figure S6. Forest and funnel plot of DASH score for joint replacement at 1 month.


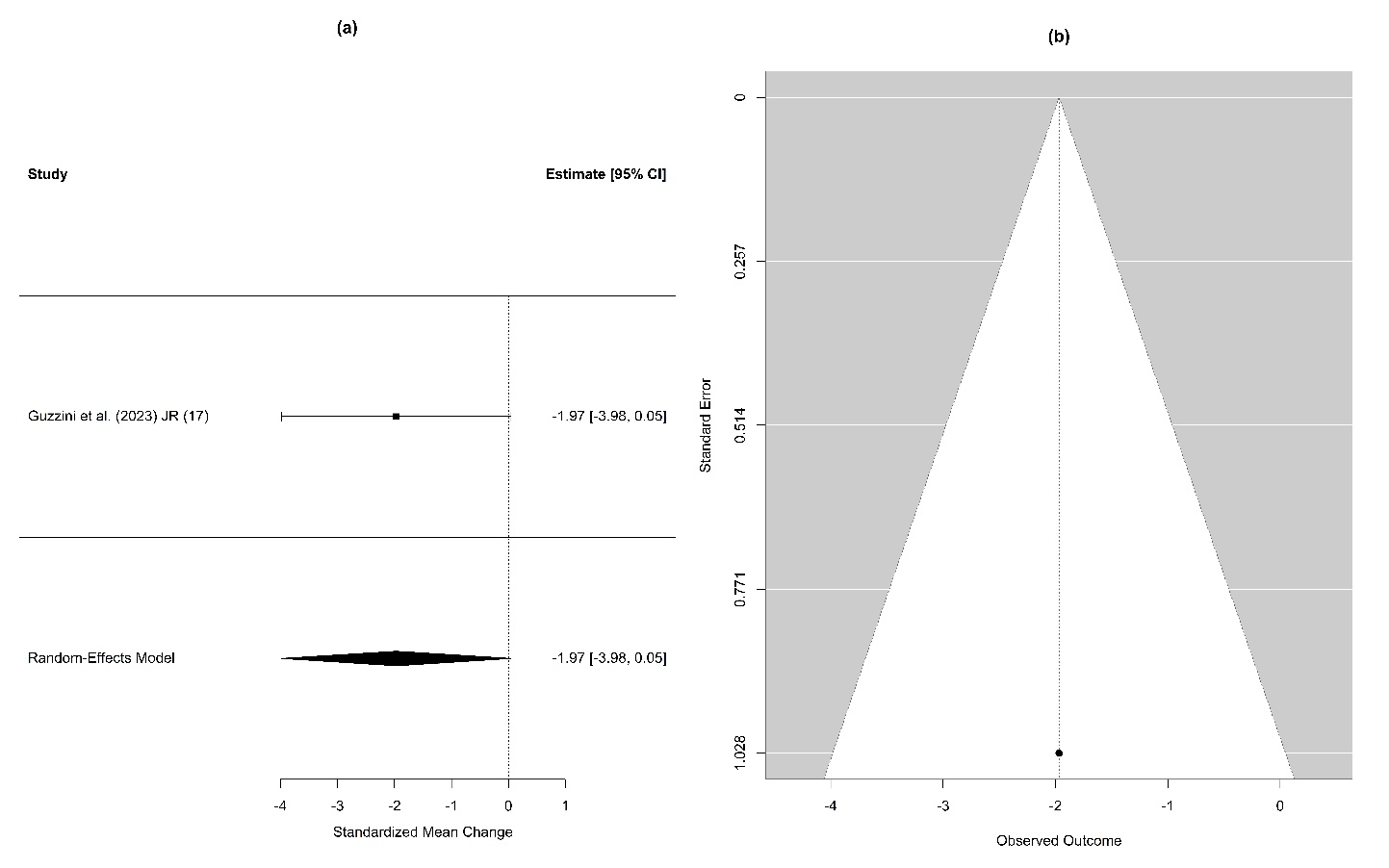


Figure S7. Forest and funnel plot of DASH score for joint replacement at 3 months.


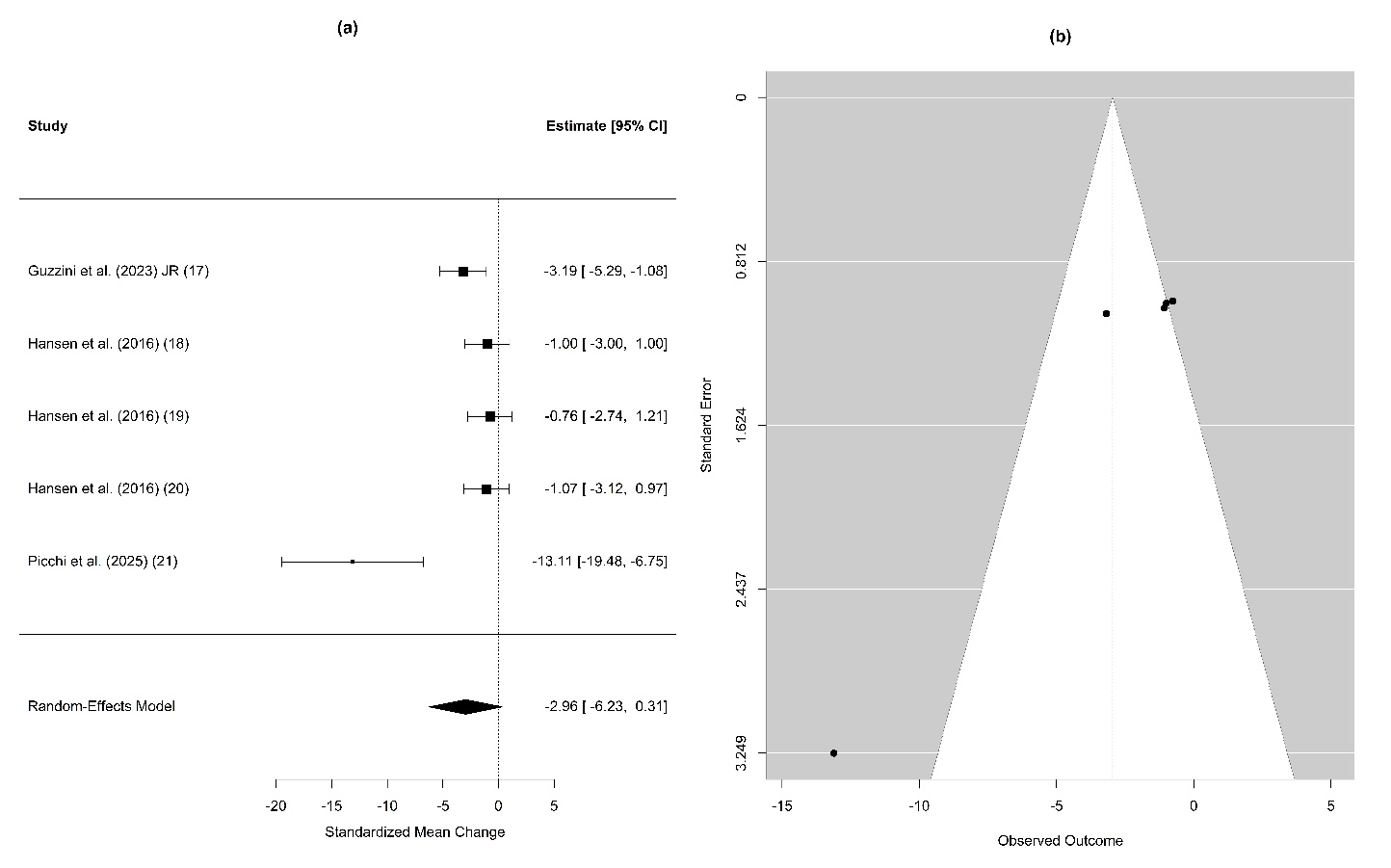


Figure S8. Forest and funnel plot of DASH score for joint replacement at 6 months.


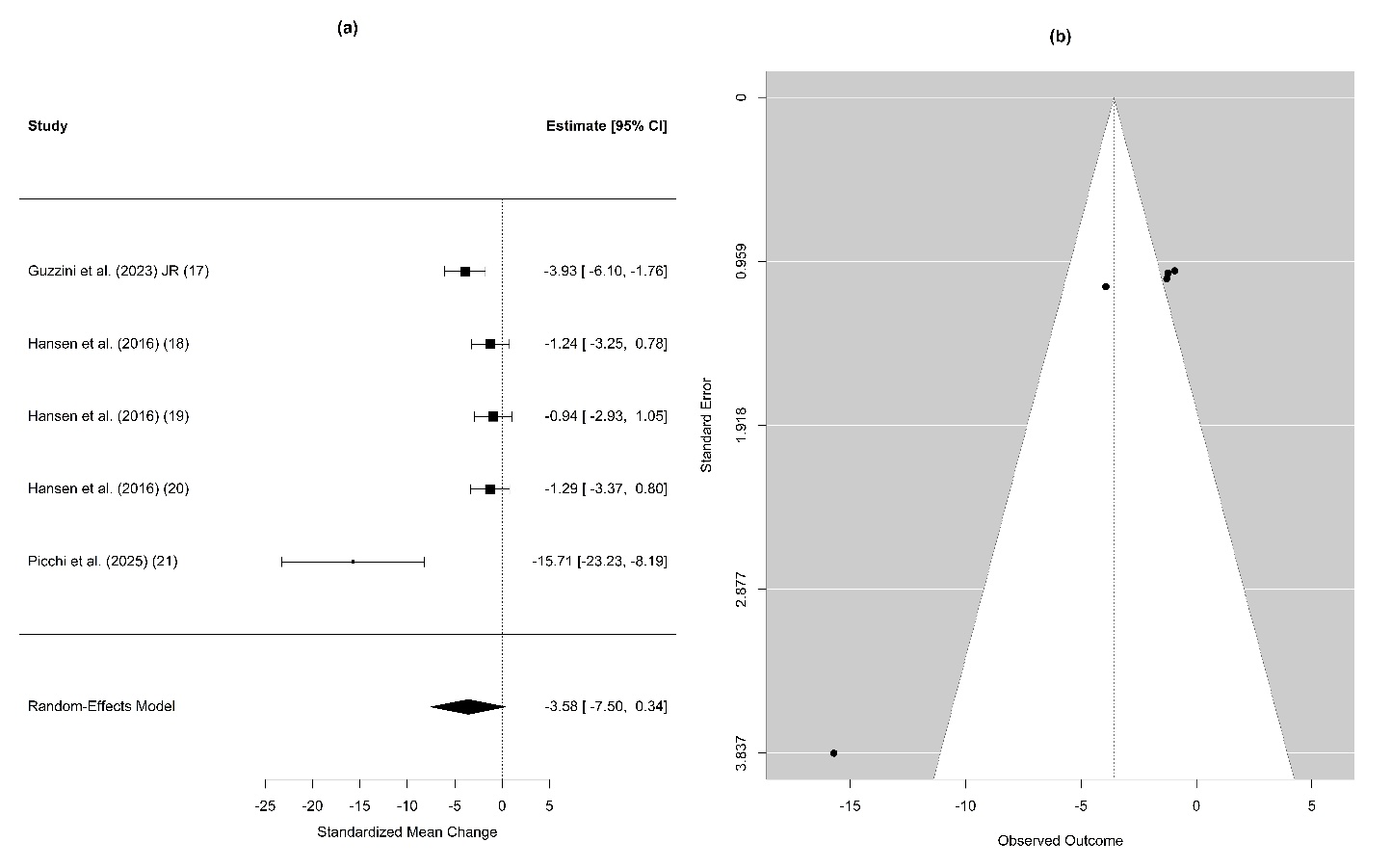


Figure S9. Forest and funnel plot of DASH score for joint replacement at 12 months.


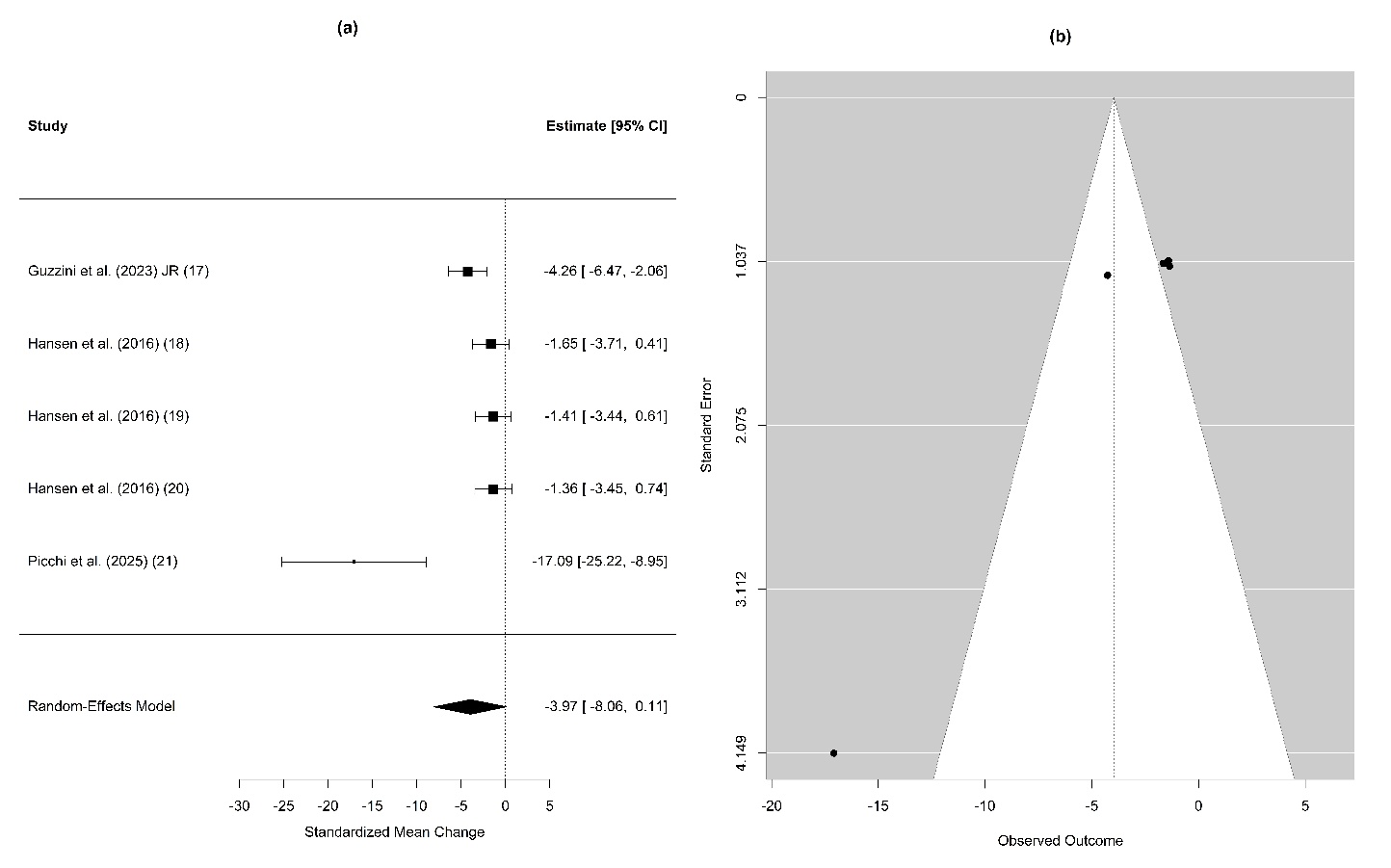


Figure S10. Forest and funnel plot of grip strength for trapeziectomy at 1 month.


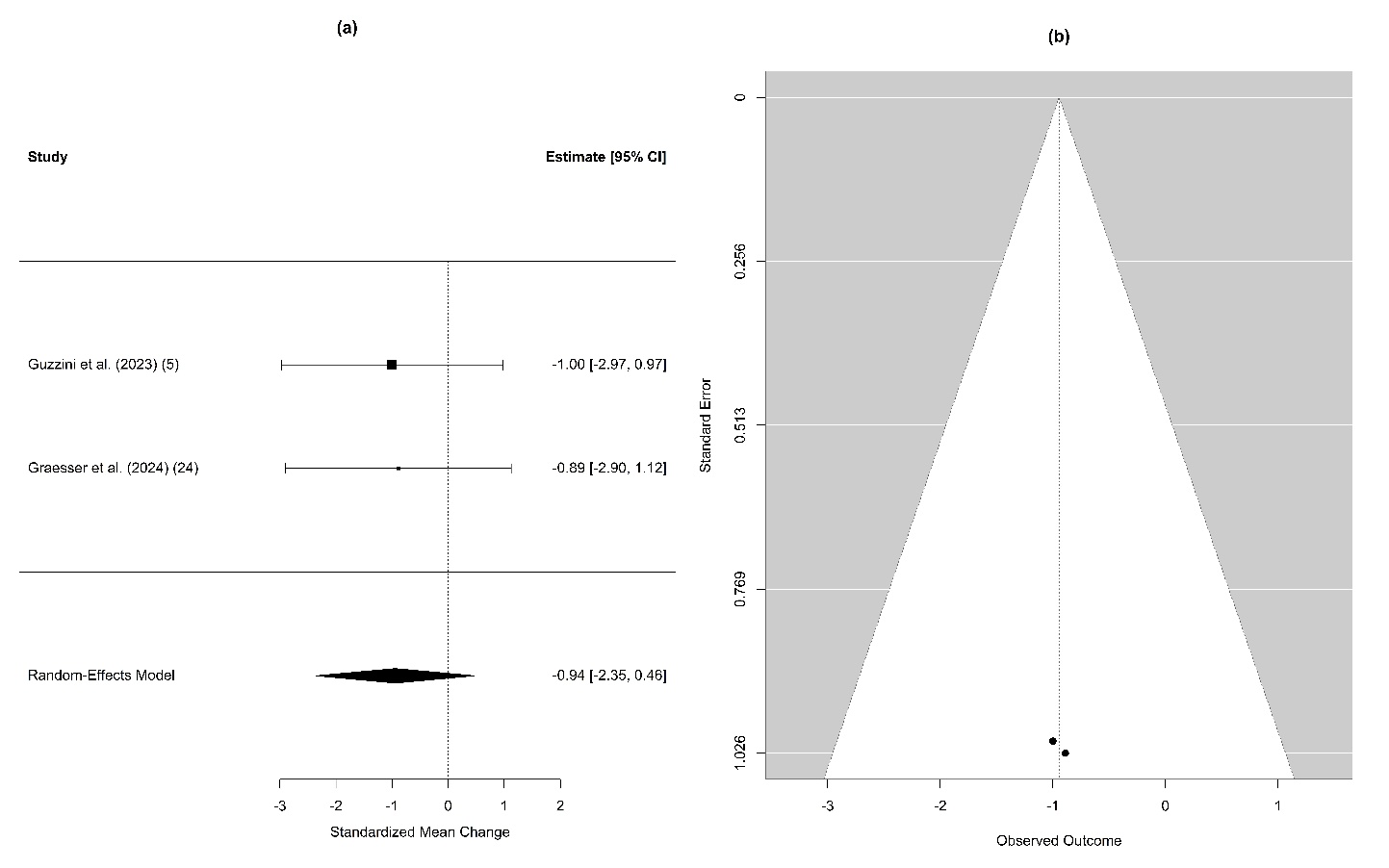


Figure S11. Forest and funnel plot of grip strength for trapeziectomy at 3 months.


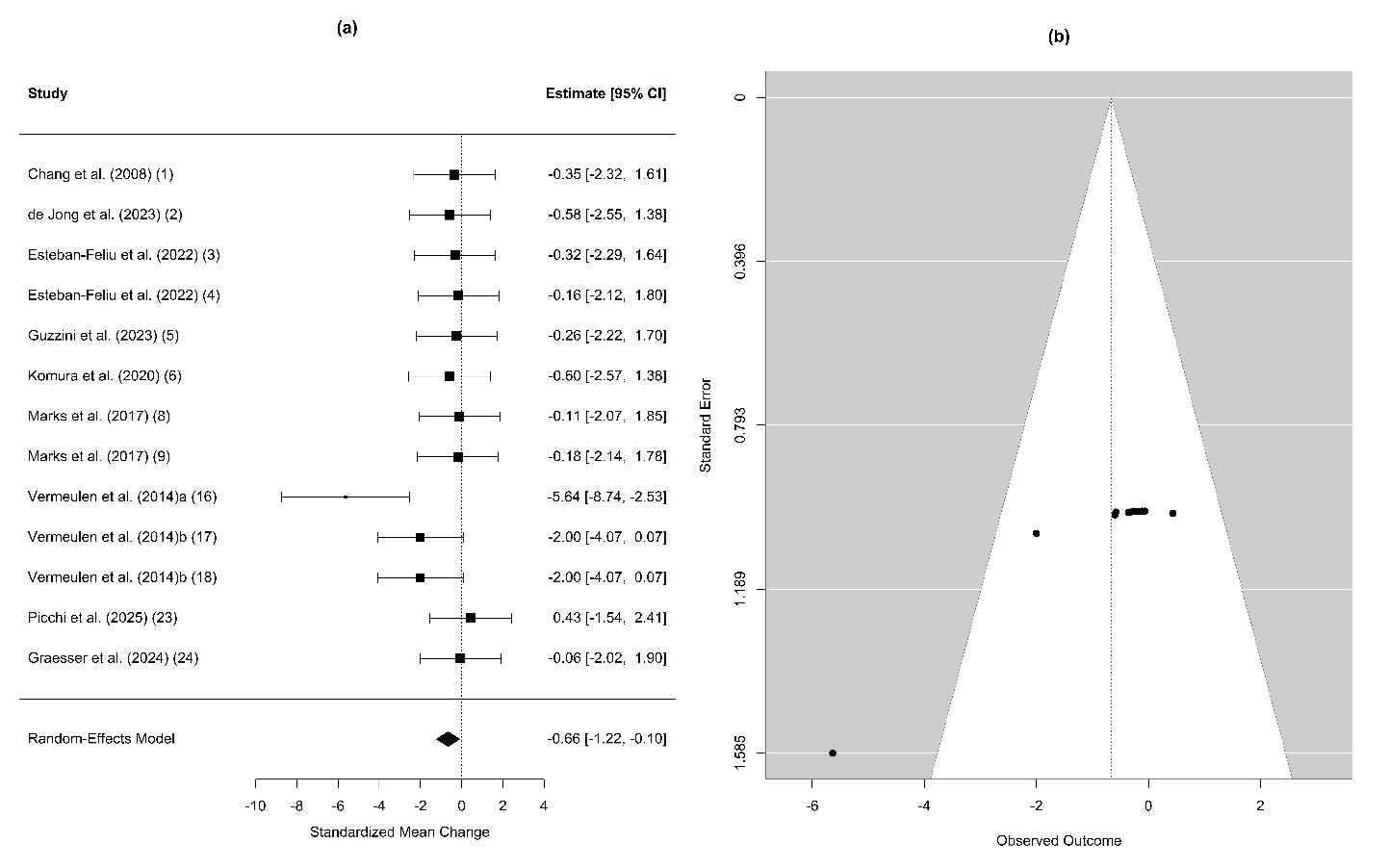


Figure S12. Forest and funnel plot of grip strength for trapeziectomy at 6 months.


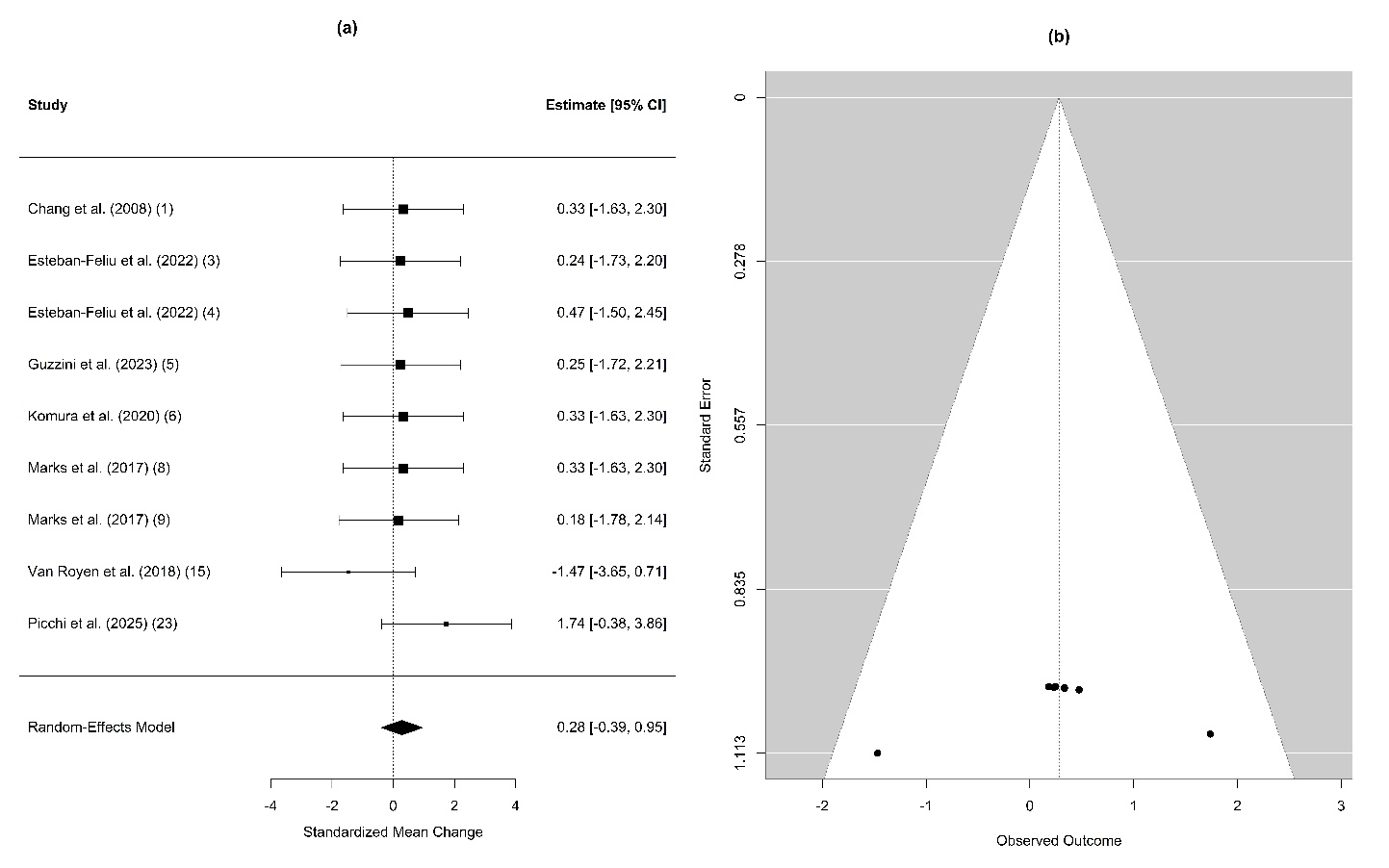


Figure S13. Forest and funnel plot of grip strength for trapeziectomy at 12 months.


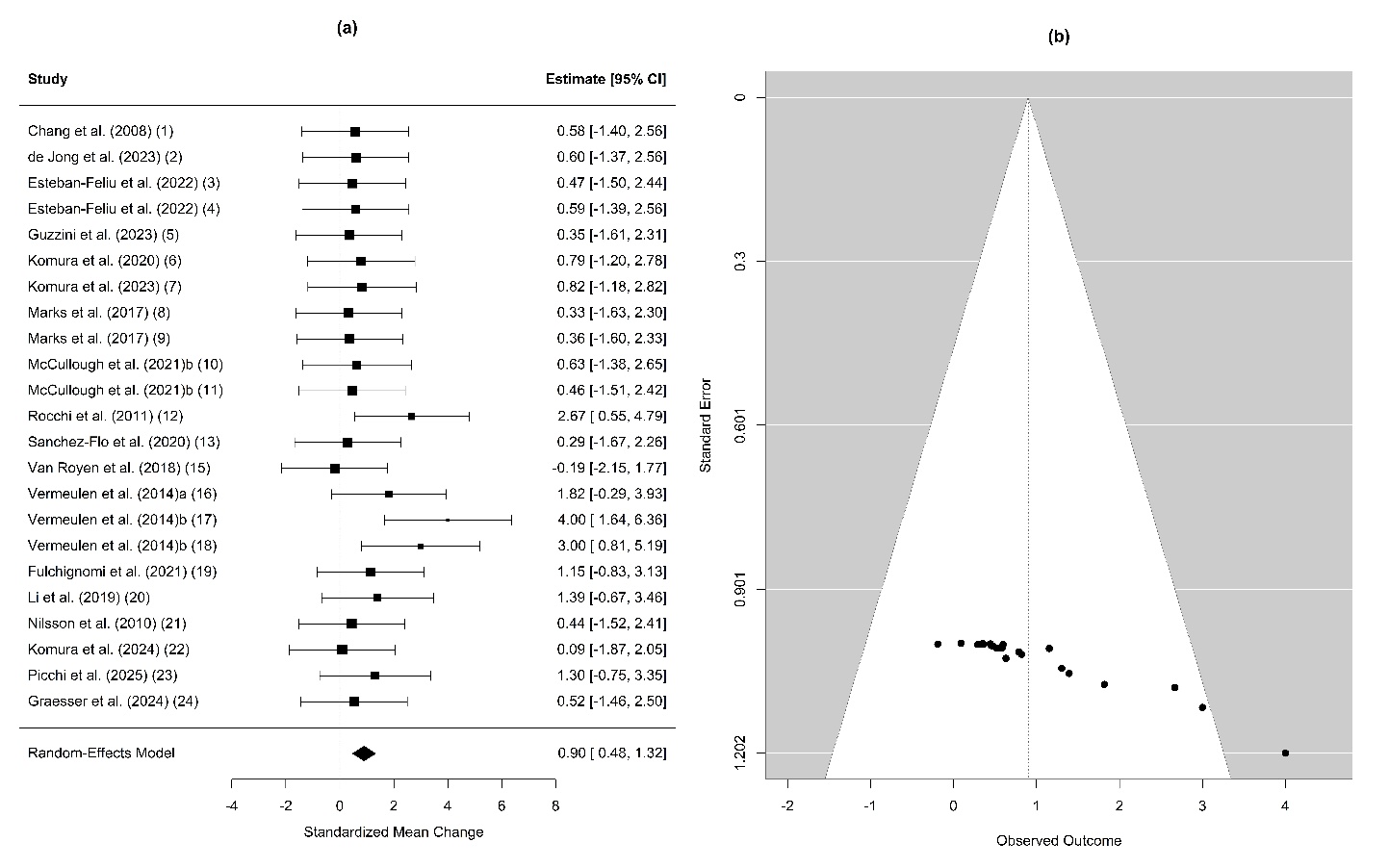


Figure S14. Forest and funnel plot of grip strength for joint replacement at 1 month.


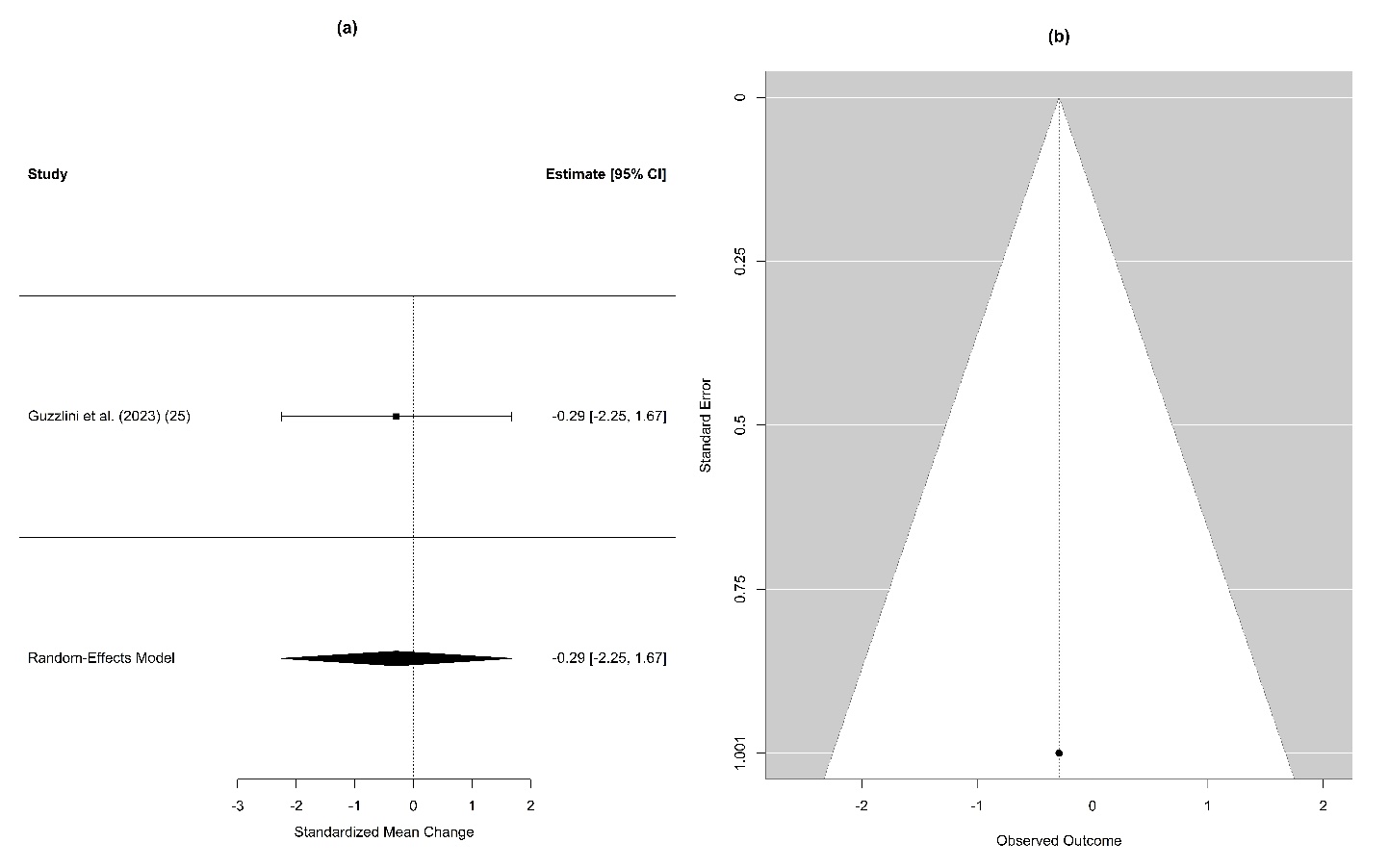


Figure S15. Forest and funnel plot of grip strength for joint replacement 3 months.


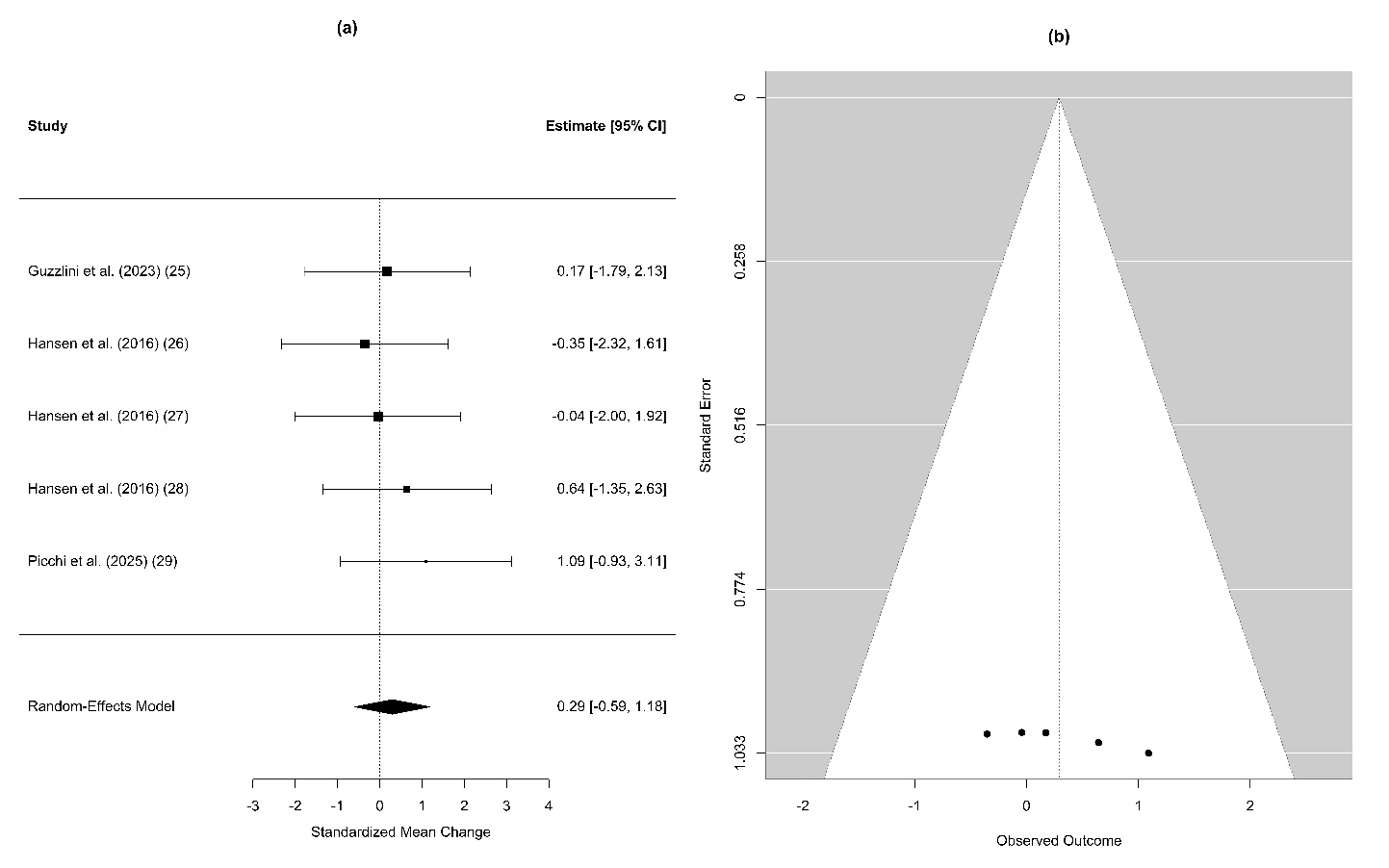


Figure S16. Forest and funnel plot of grip strength for joint replacement at 6 months.


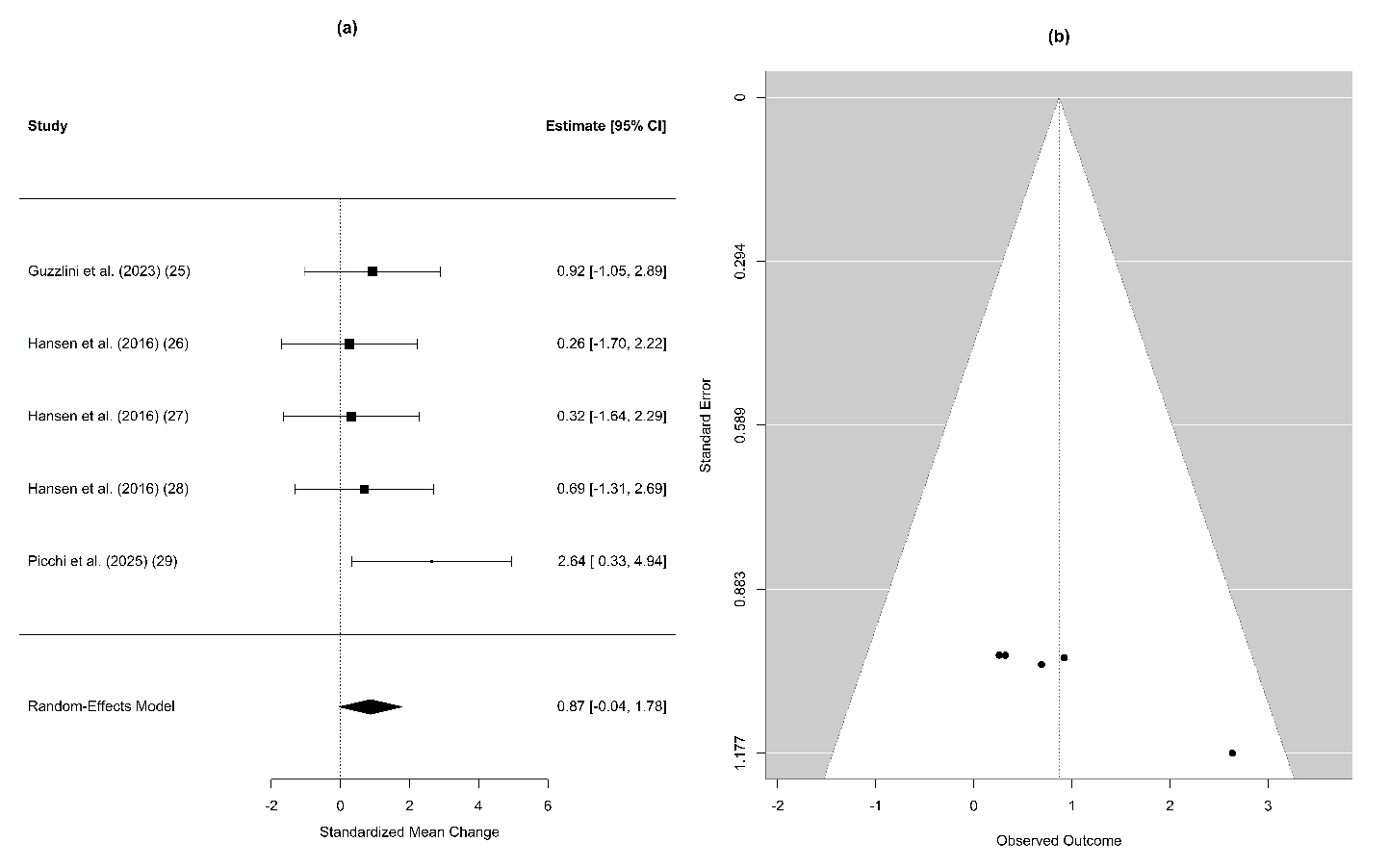


Figure S17. Forest and funnel plot of grip strength for joint replacement at 12 months.


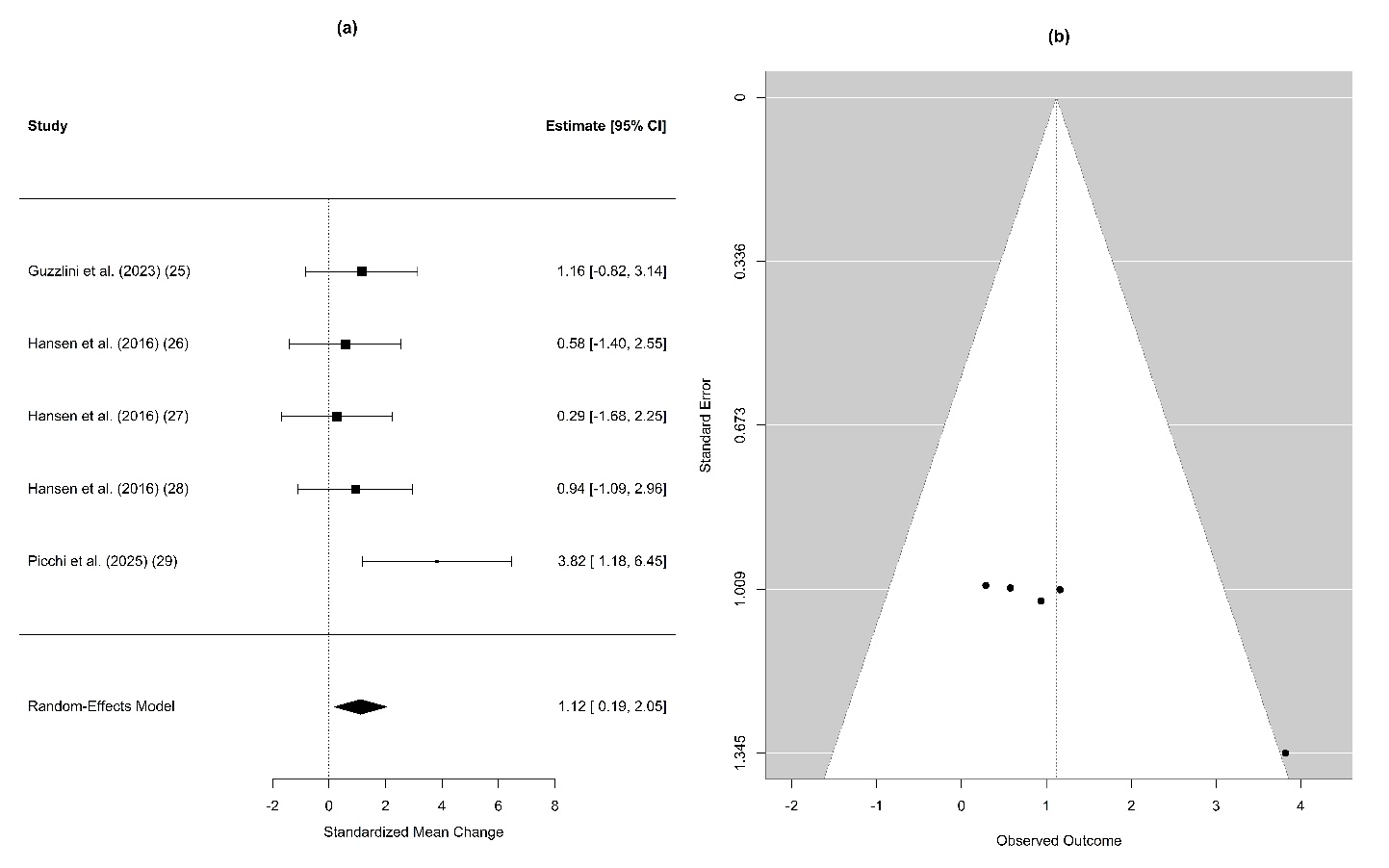


Figure S18. Forest and funnel plot of key pinch strength for trapeziectomy at 1 month.


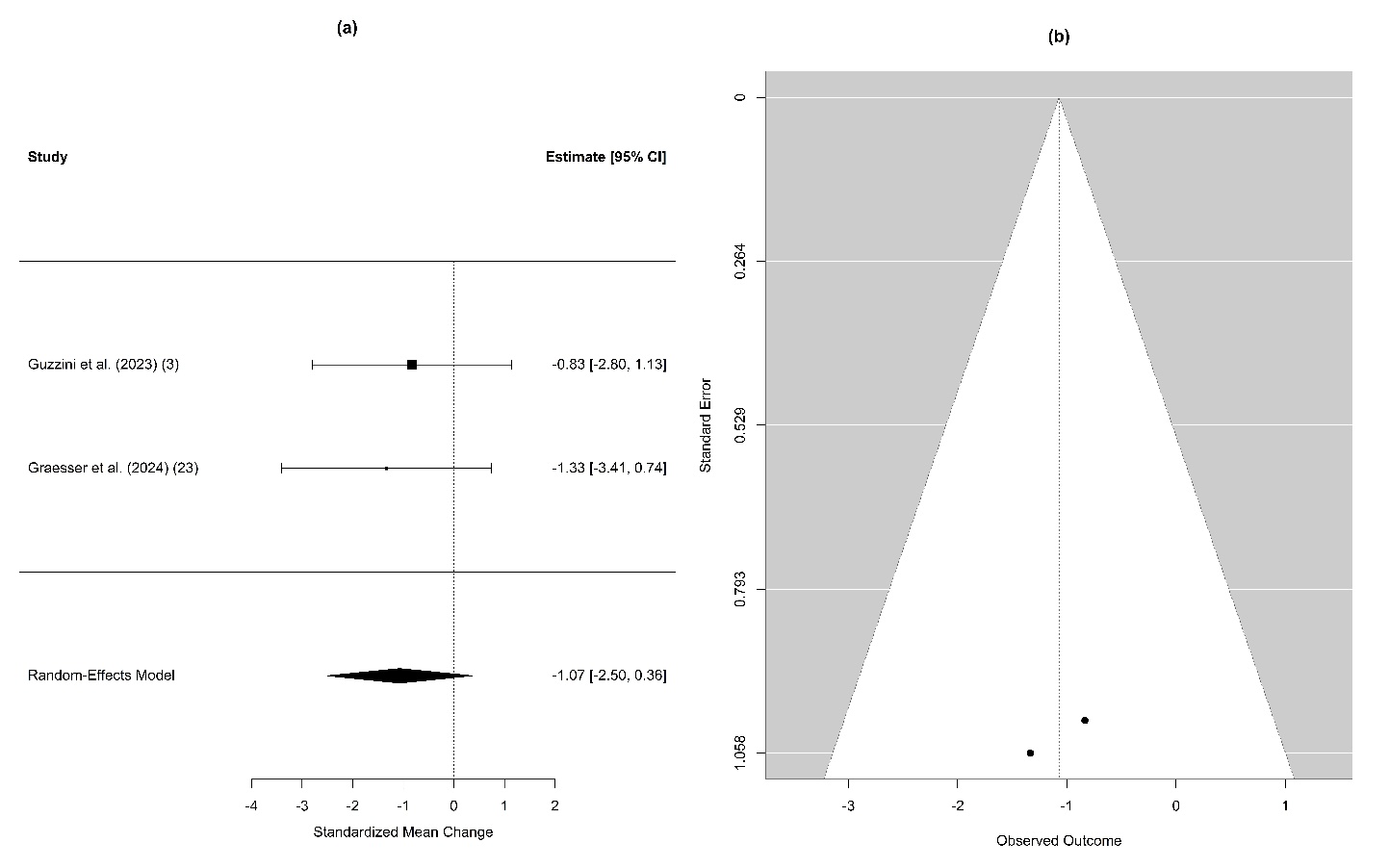


Figure S19. Forest and funnel plot of key pinch strength for trapeziectomy at 3 months.


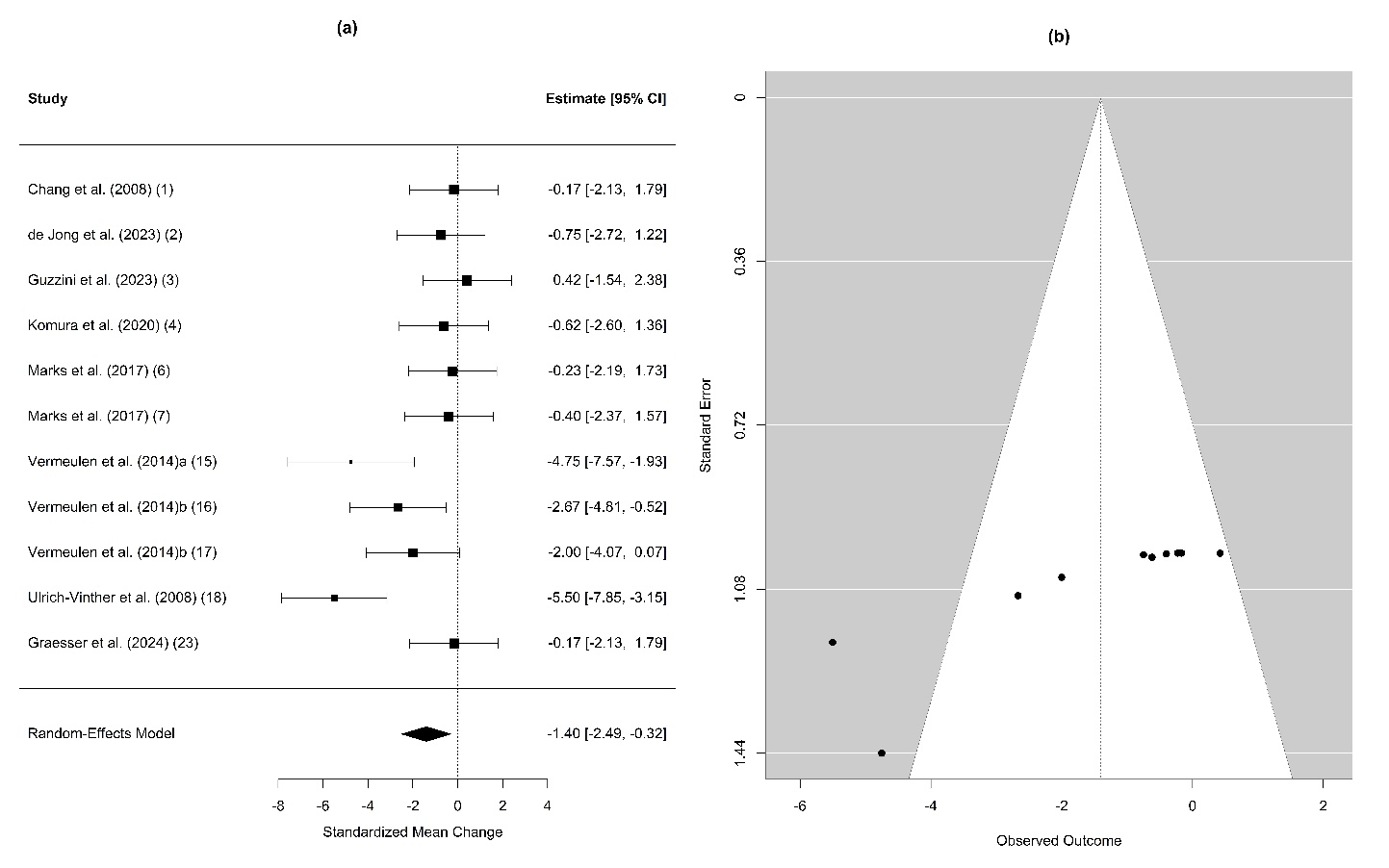


Figure S20. Forest and funnel plot of key pinch strength for trapeziectomy at 6 months.


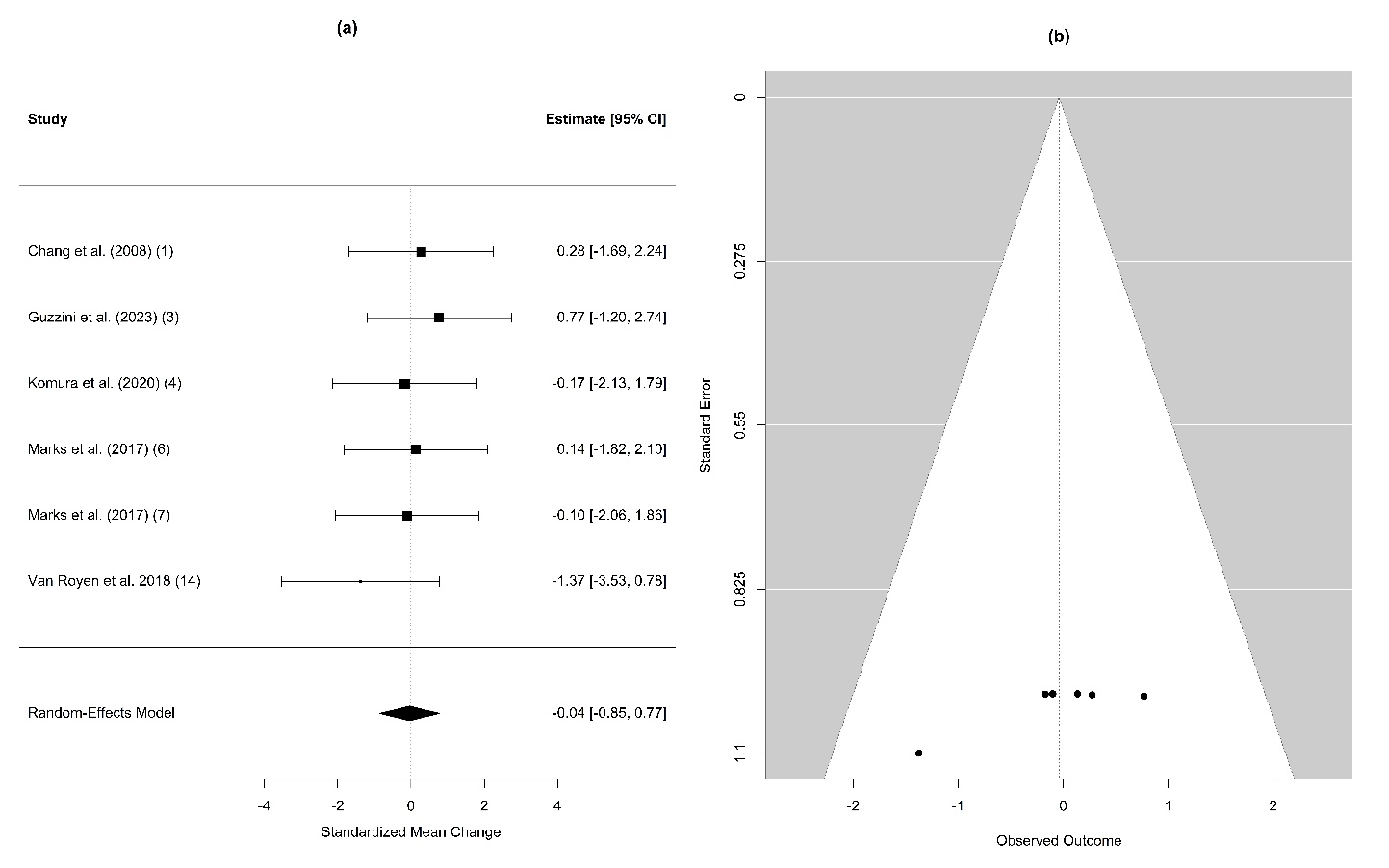


Figure S21. Forest and funnel plot of key pinch strength for trapeziectomy at 12 months.


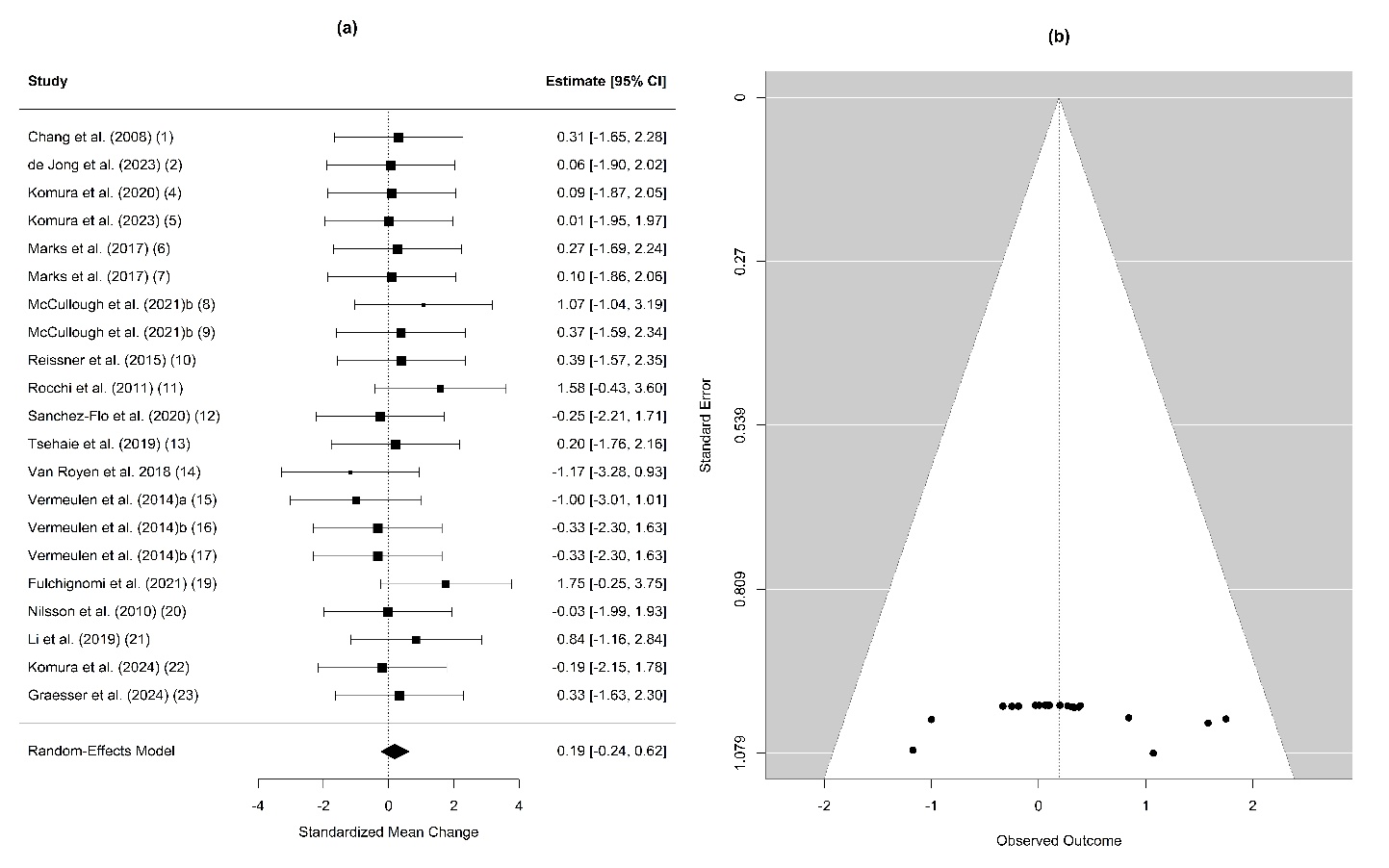


Figure S22. Forest and funnel plot of key pinch strength for joint replacement at 1 month.


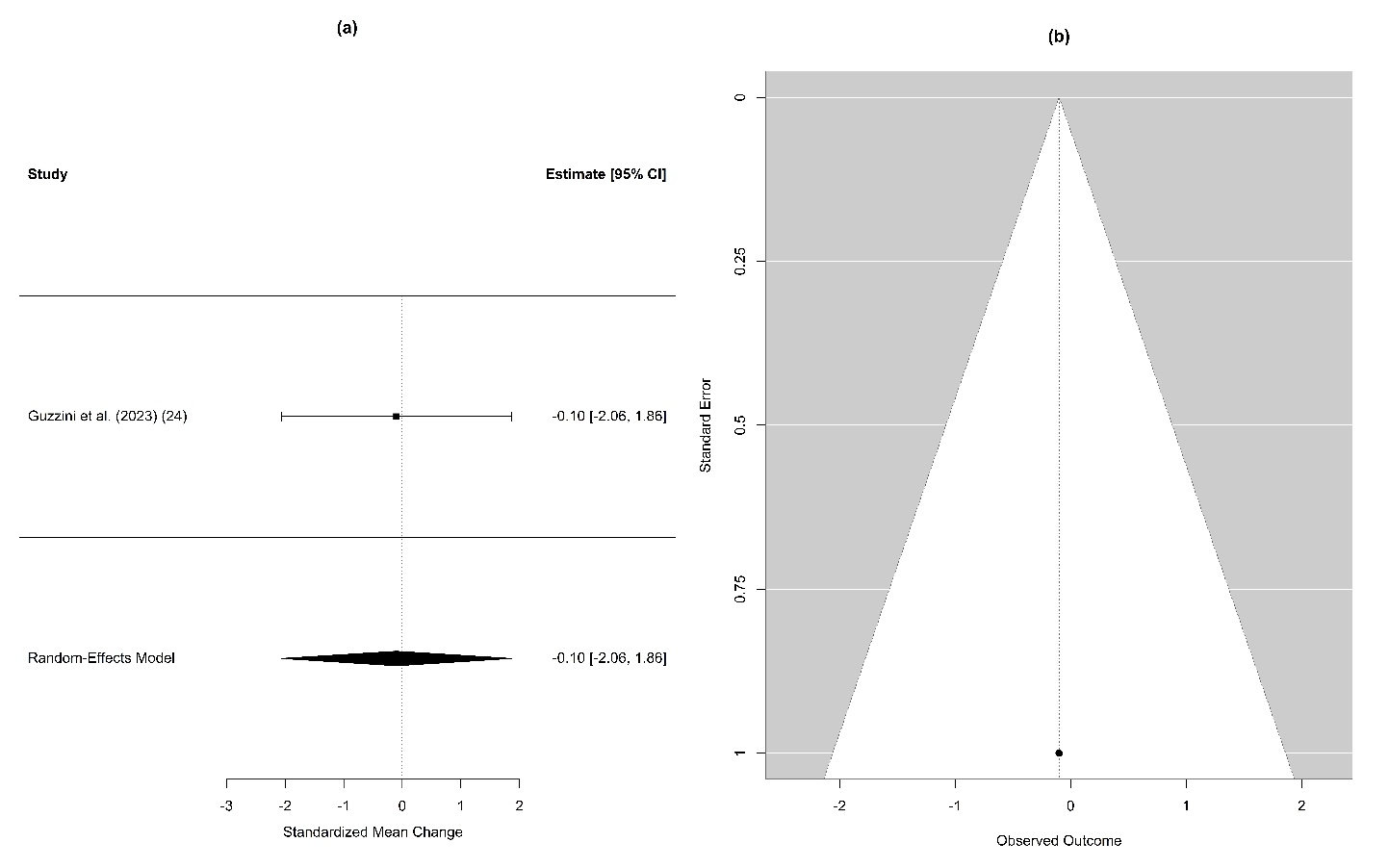


Figure S23. Forest and funnel plot of key pinch strength for joint replacement at 3 months.


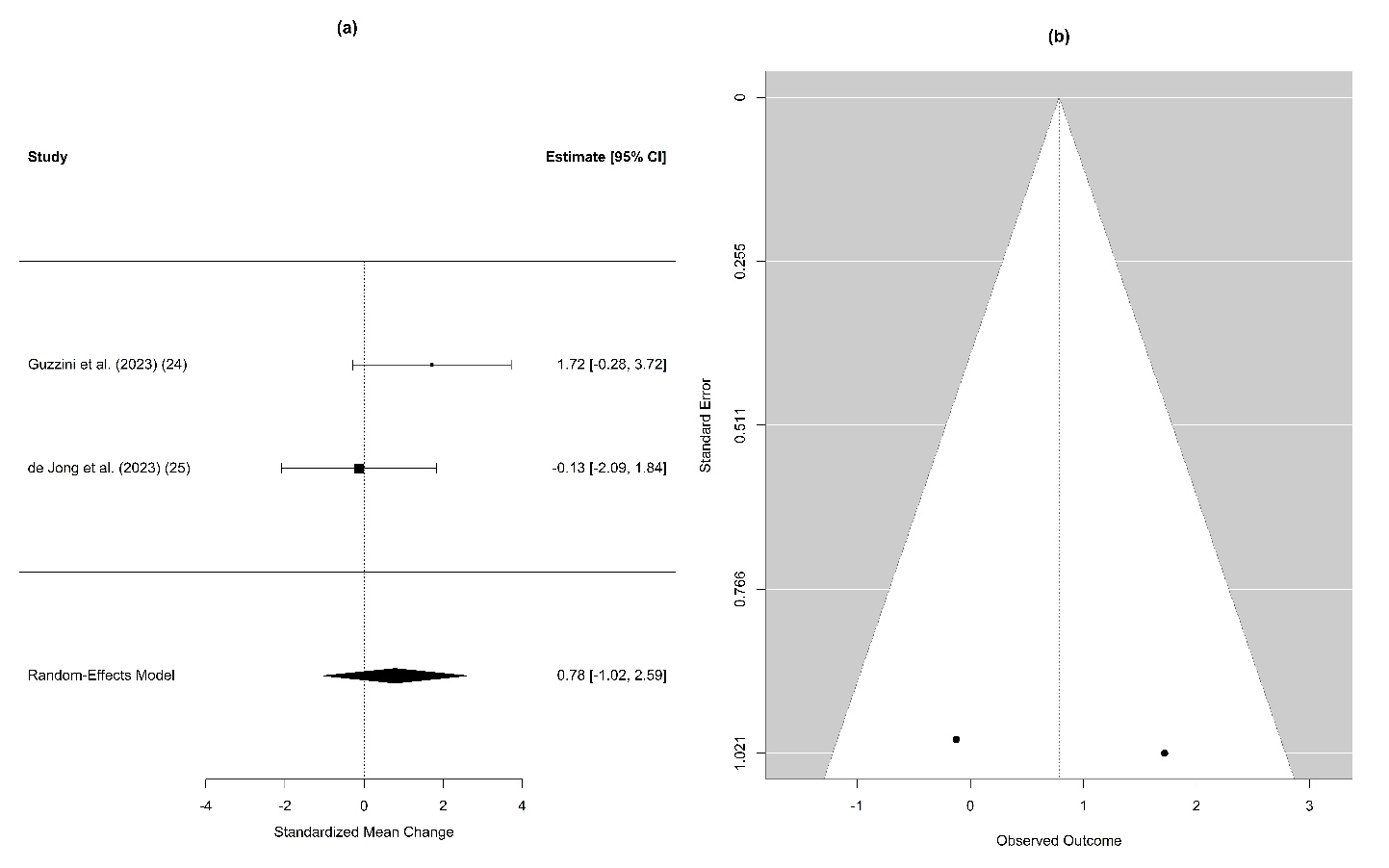


Figure S24. Forest and funnel plot of key pinch strength for joint replacement at 6 months.


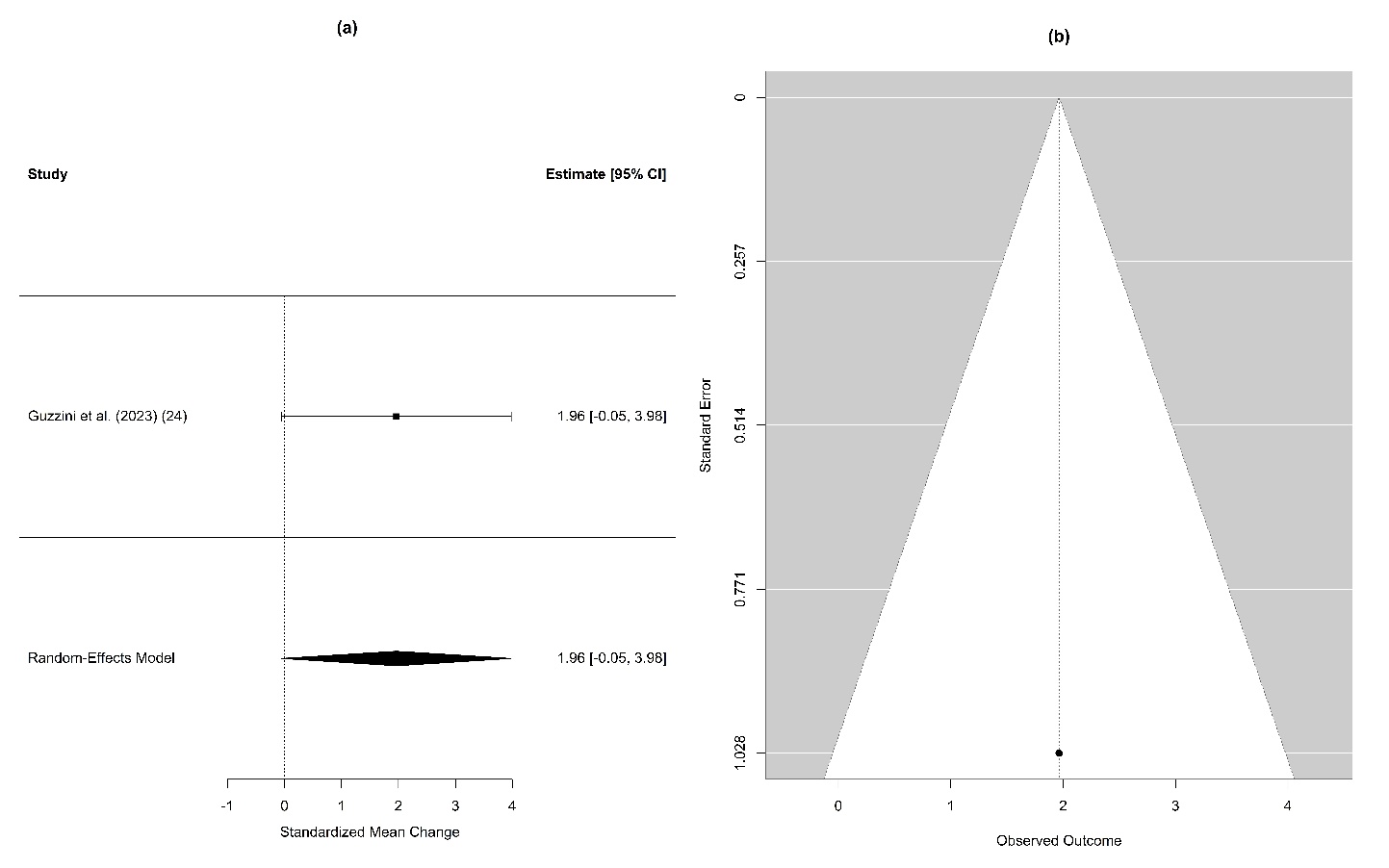


Figure S25. Forest and funnel plot of key pinch strength for joint replacement at 12 months.


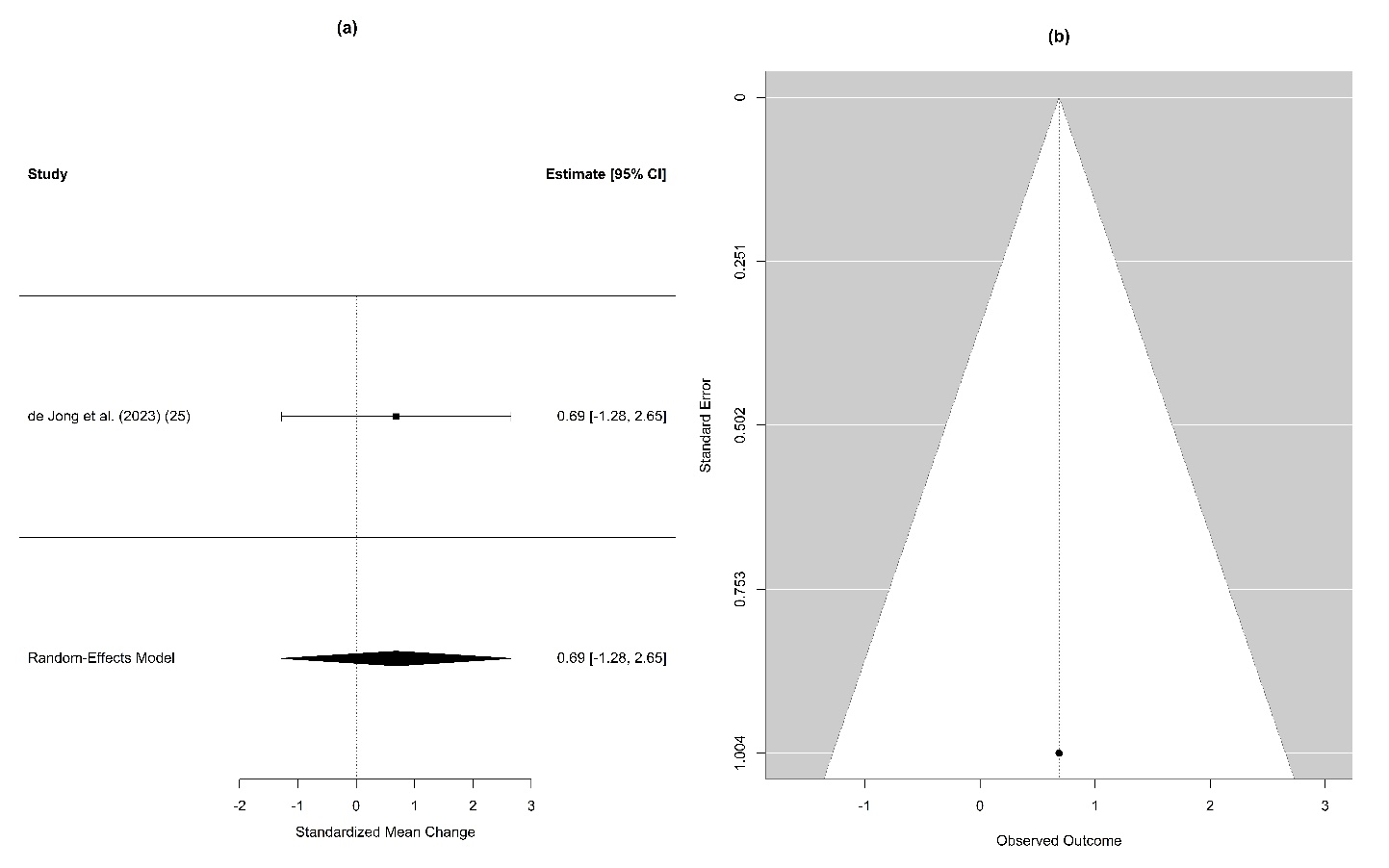


Figure S26. Cumulative SMC of DASH score for uncemented CMC joint replacement.


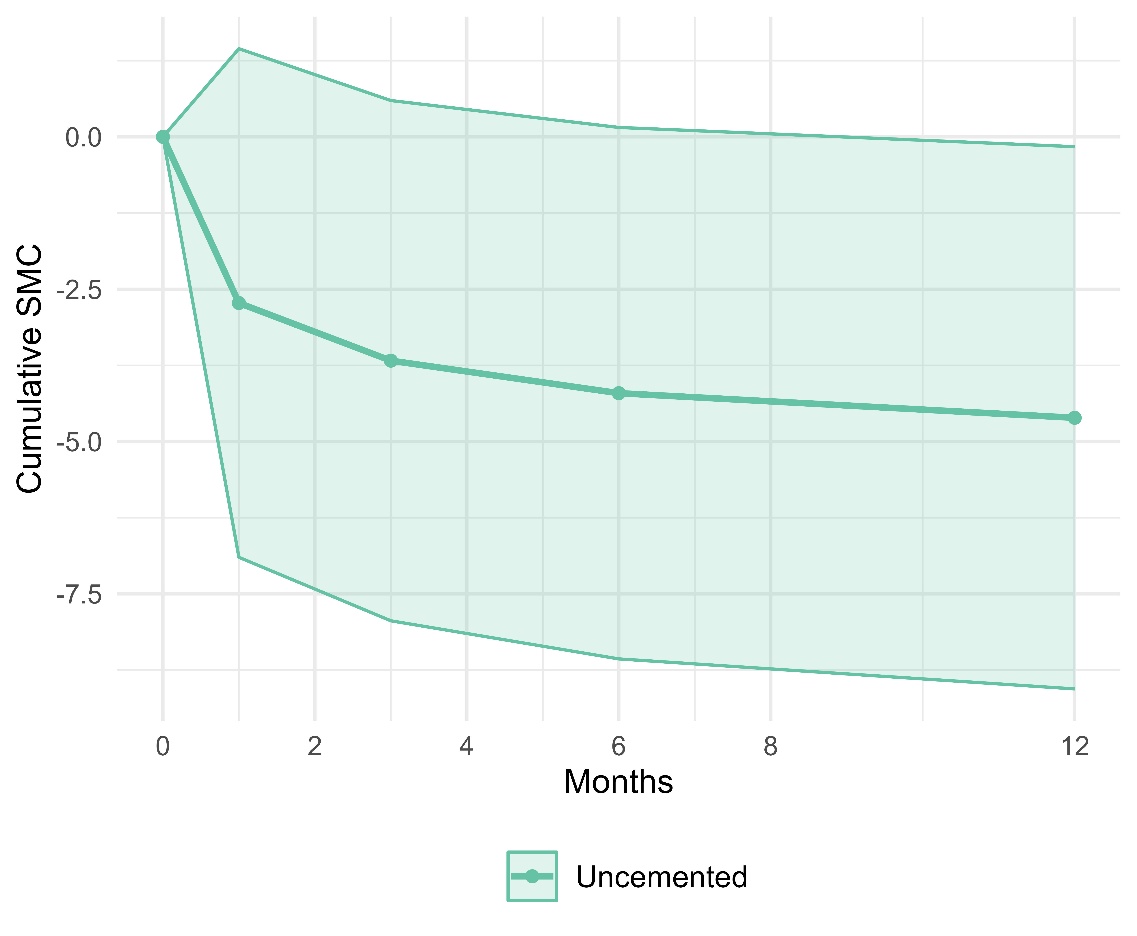


Figure S27. Cumulative SMC of grip strengths for uncemented CMC joint replacement.


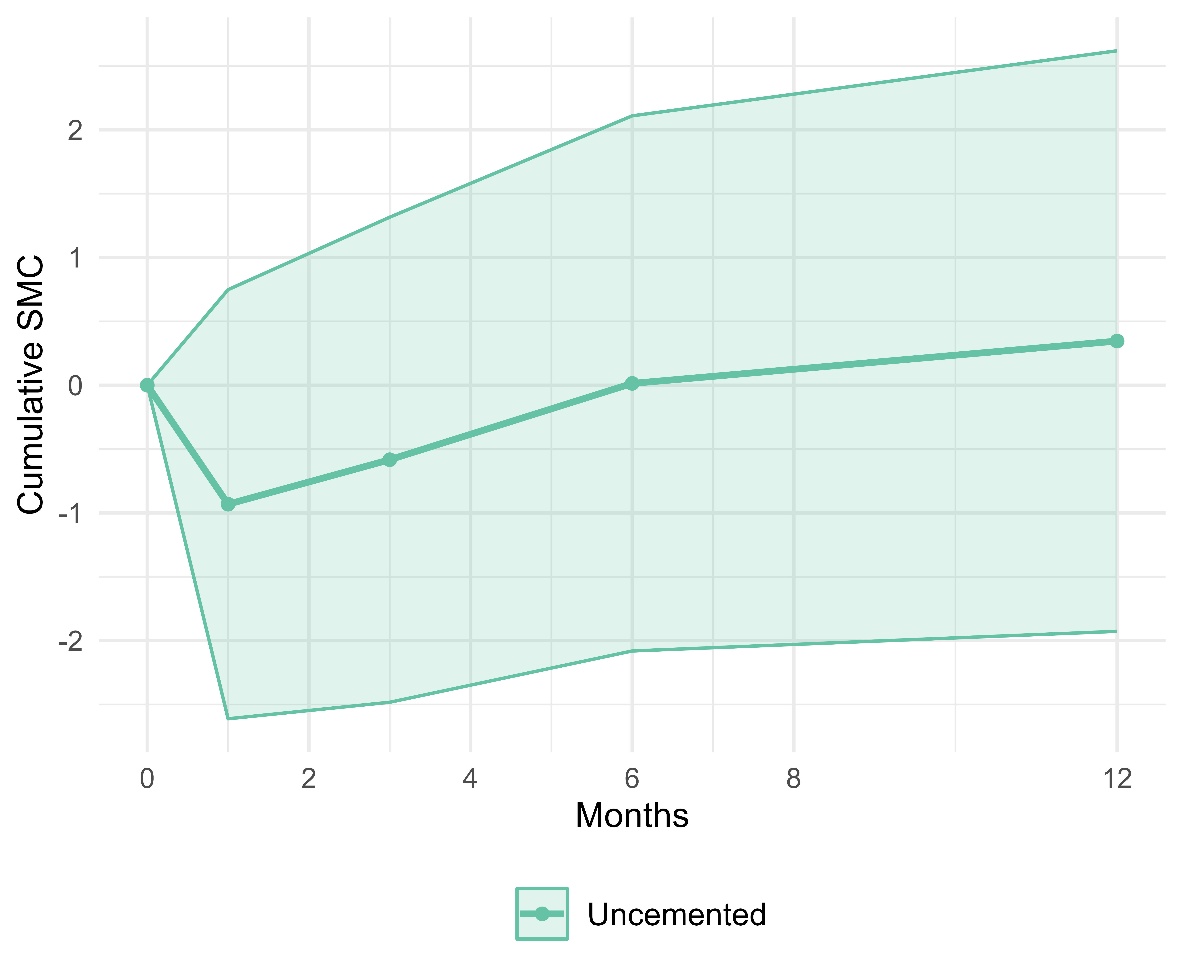


Figure S28. Cumulative SMC of key pinch strengths for uncemented CMC joint replacement.


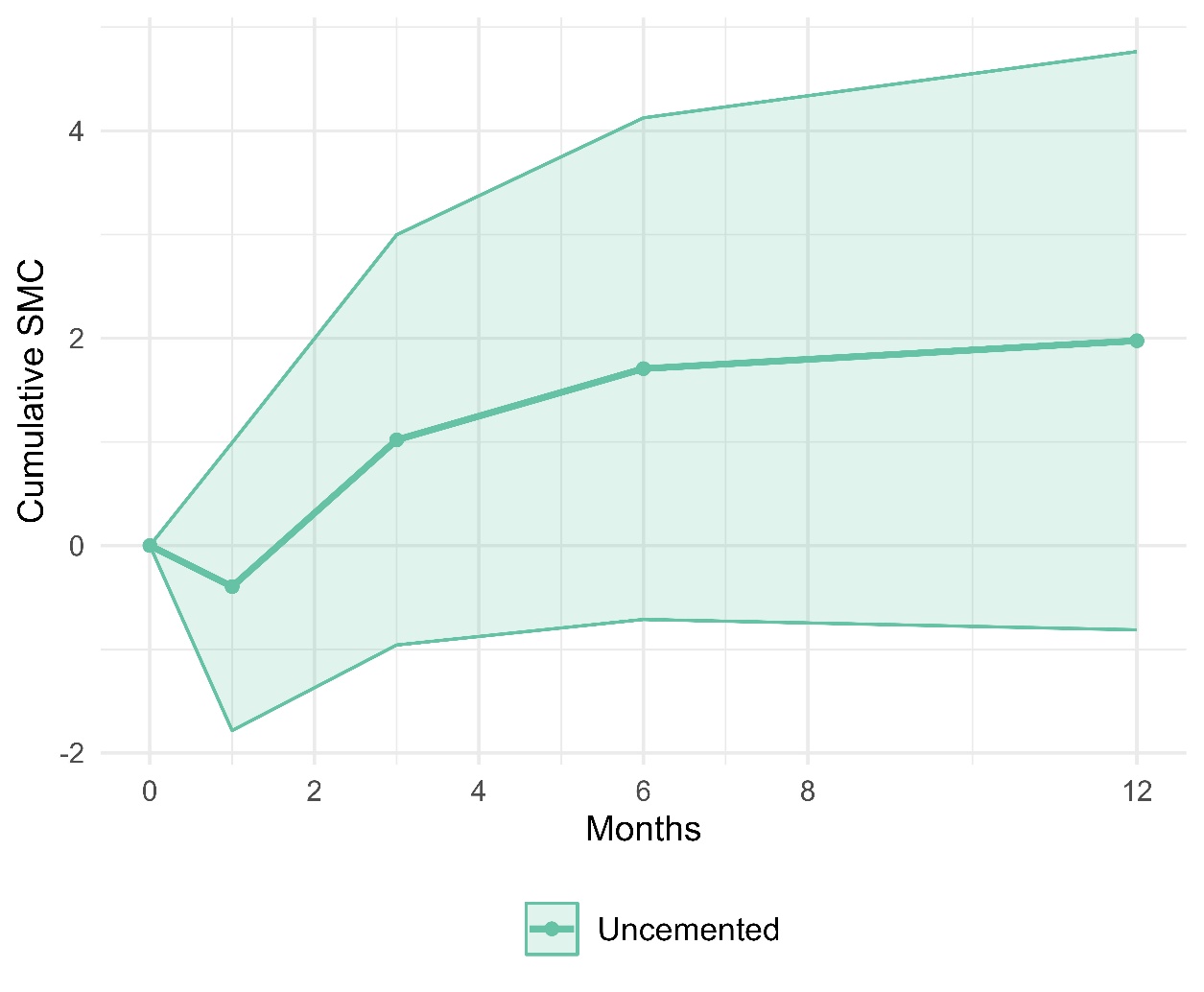


Figure S29. Cumulative SMC of grip strengths for dual mobility vs single mobility CMC joint replacement.


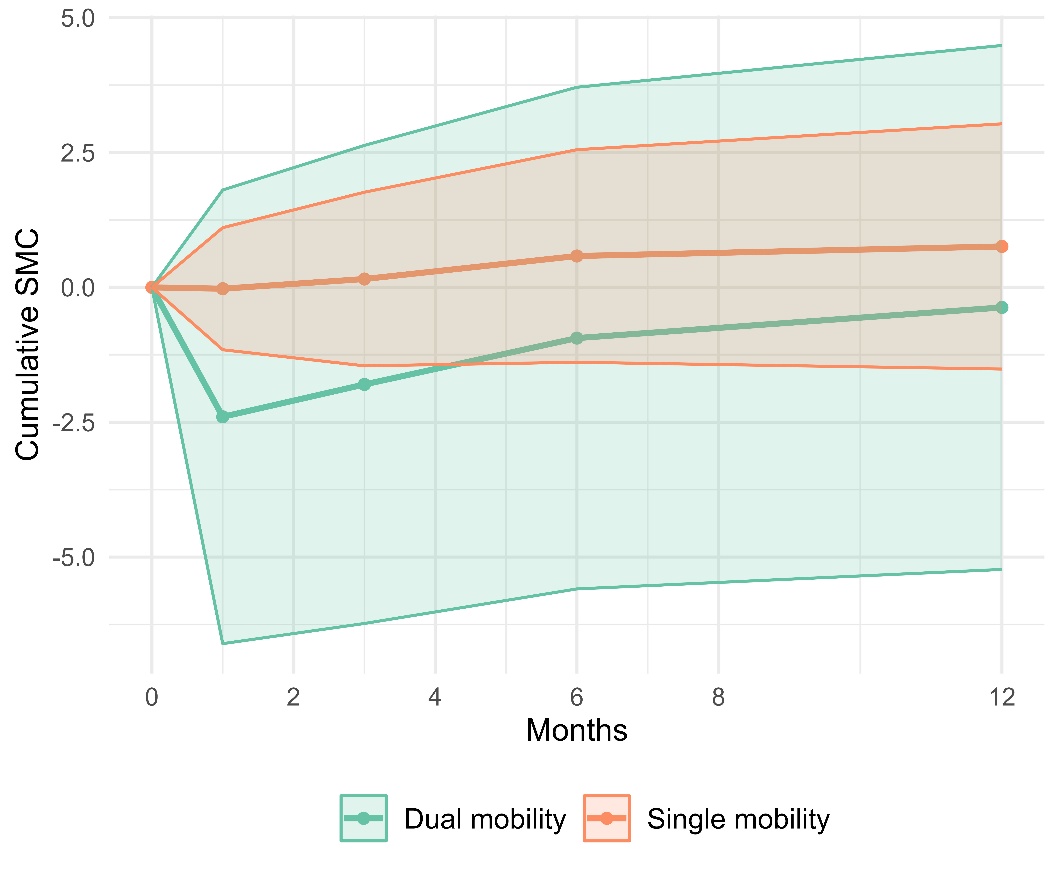


Figure S30. Cumulative SMC of key pinch strengths for dual mobility vs single mobility CMC joint replacement.


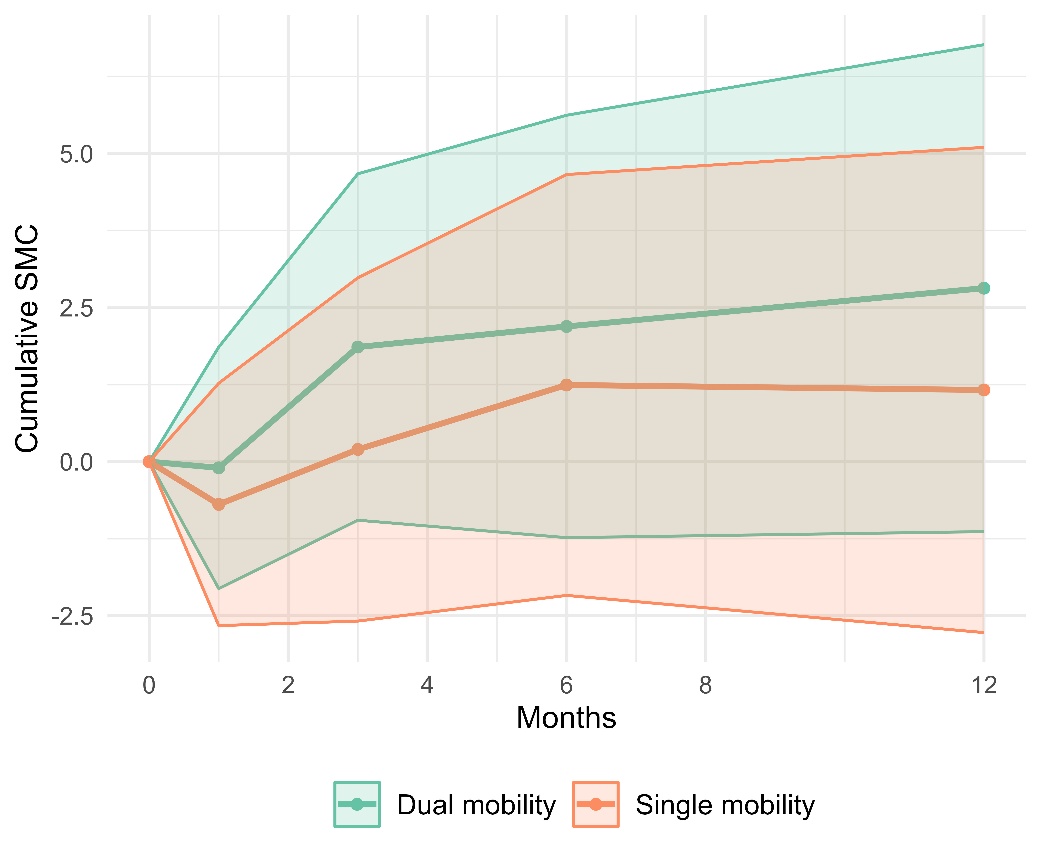


Figure S31. Cumulative SMC of DASH score for immobilisation protocol for trapeziectomy and CMC joint replacement.


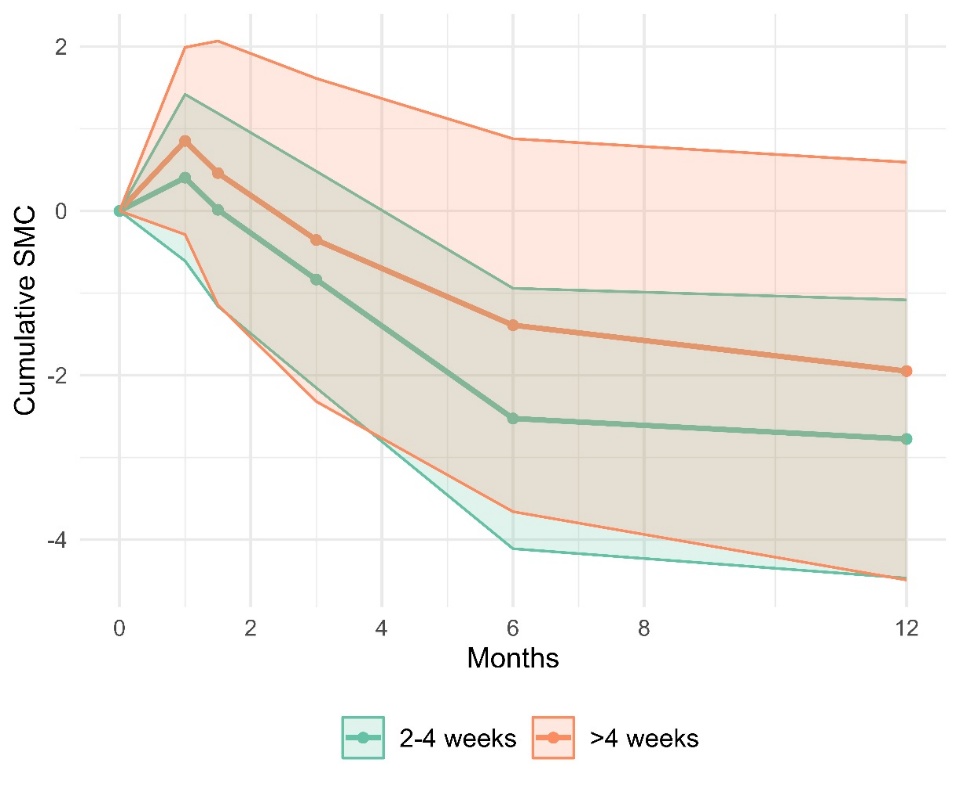


Figure S32. Cumulative SMC of grip strengths for immobilisation protocol for trapeziectomy and CMC joint replacement.


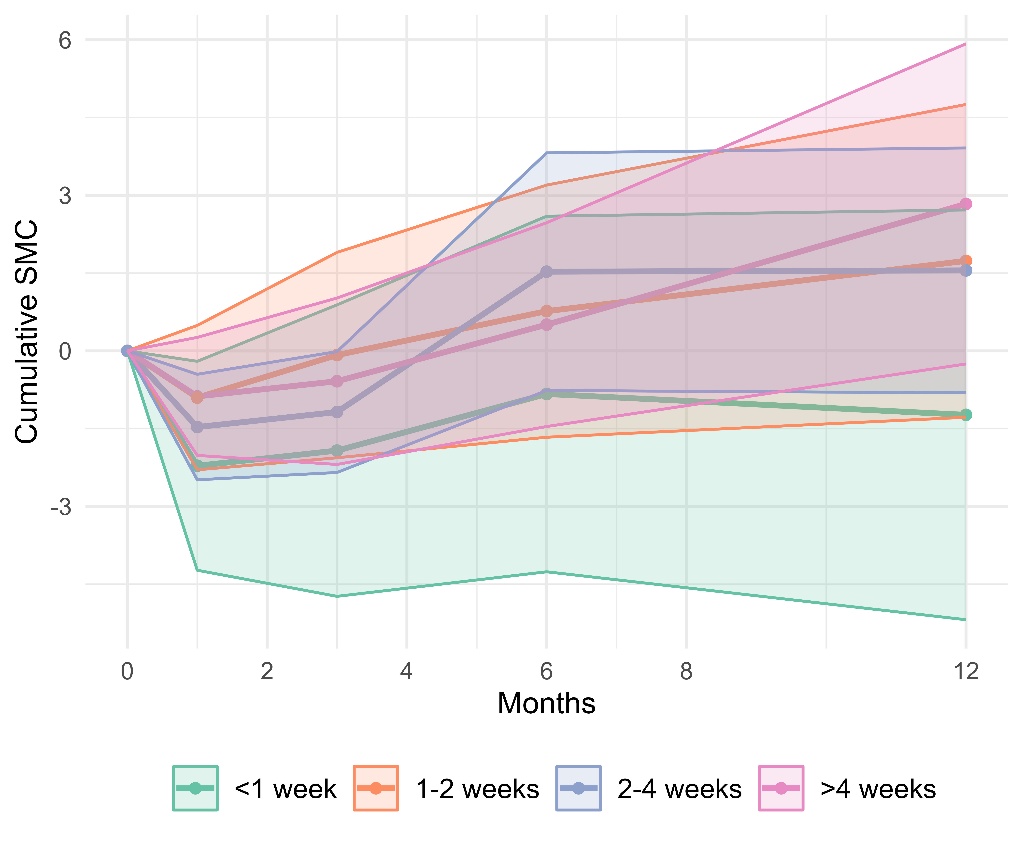


Figure S33. Cumulative SMC of key pinch strengths for immobilisation protocol for trapeziectomy and CMC joint replacement.


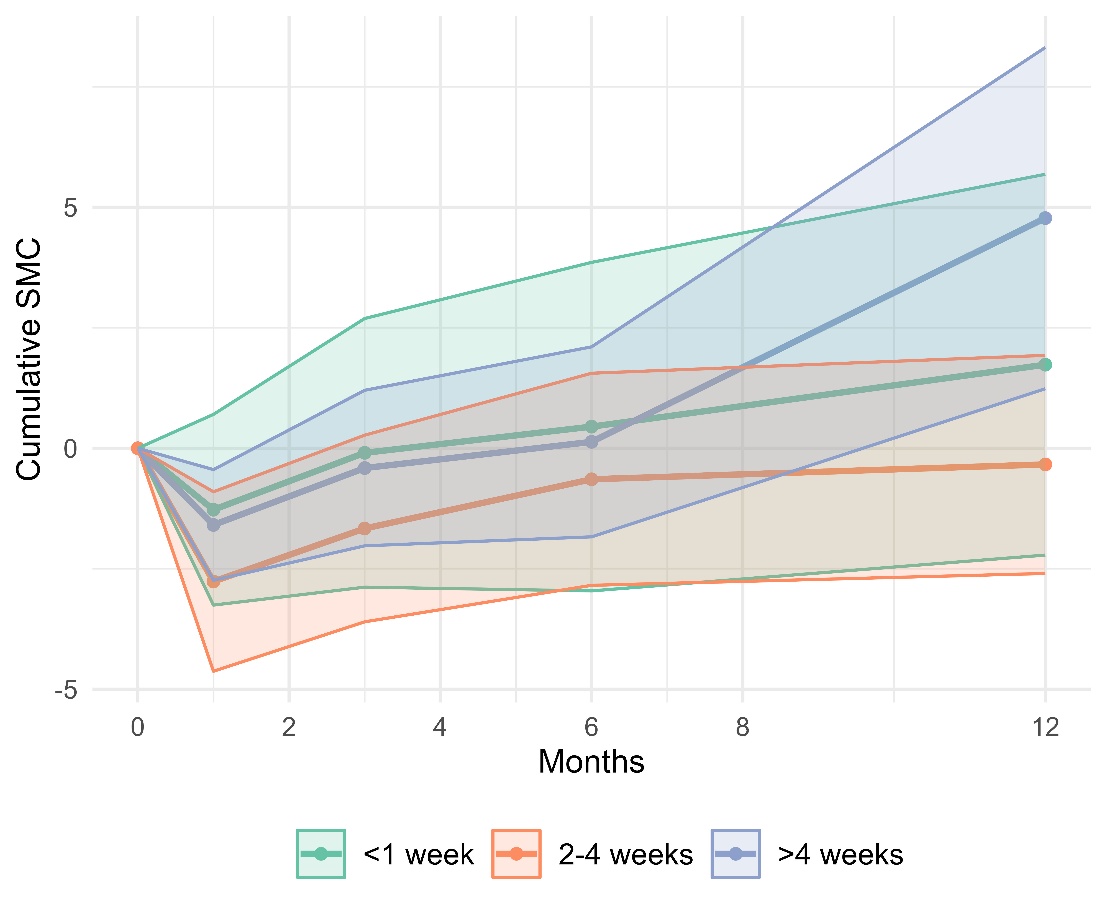


Figure S34. Cumulative SMC of DASH score for types of trapeziectomy.


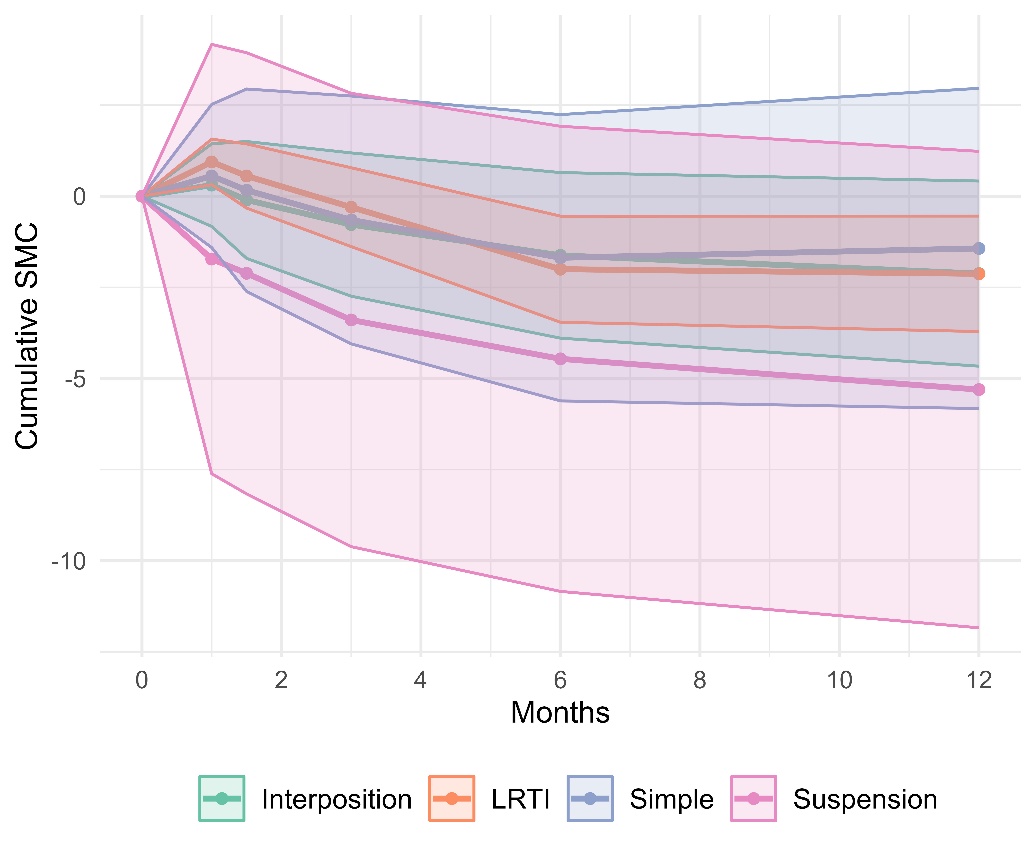


Figure S35. Cumulative SMC of grip strengths for types of trapeziectomy.


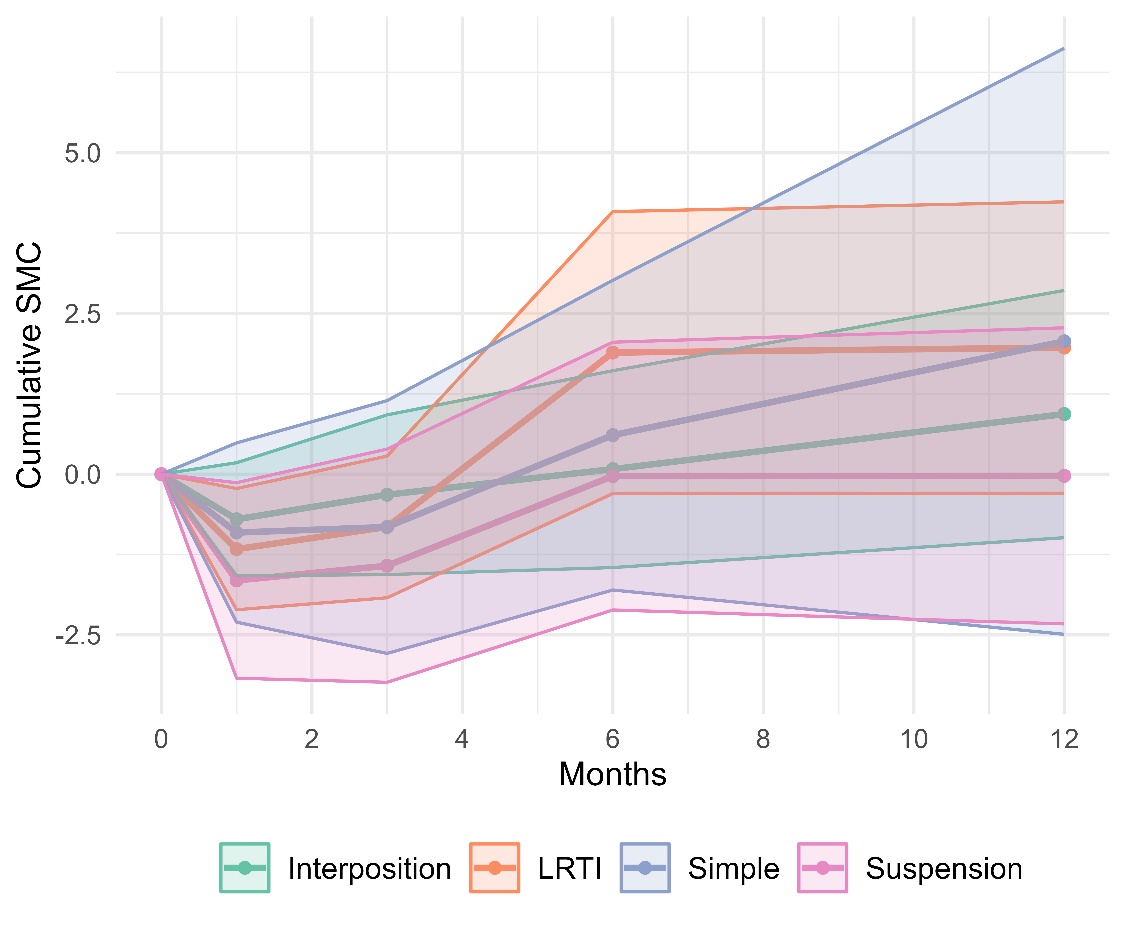


Figure S36. Cumulative SMC of key pinch strengths for types of trapeziectomy.


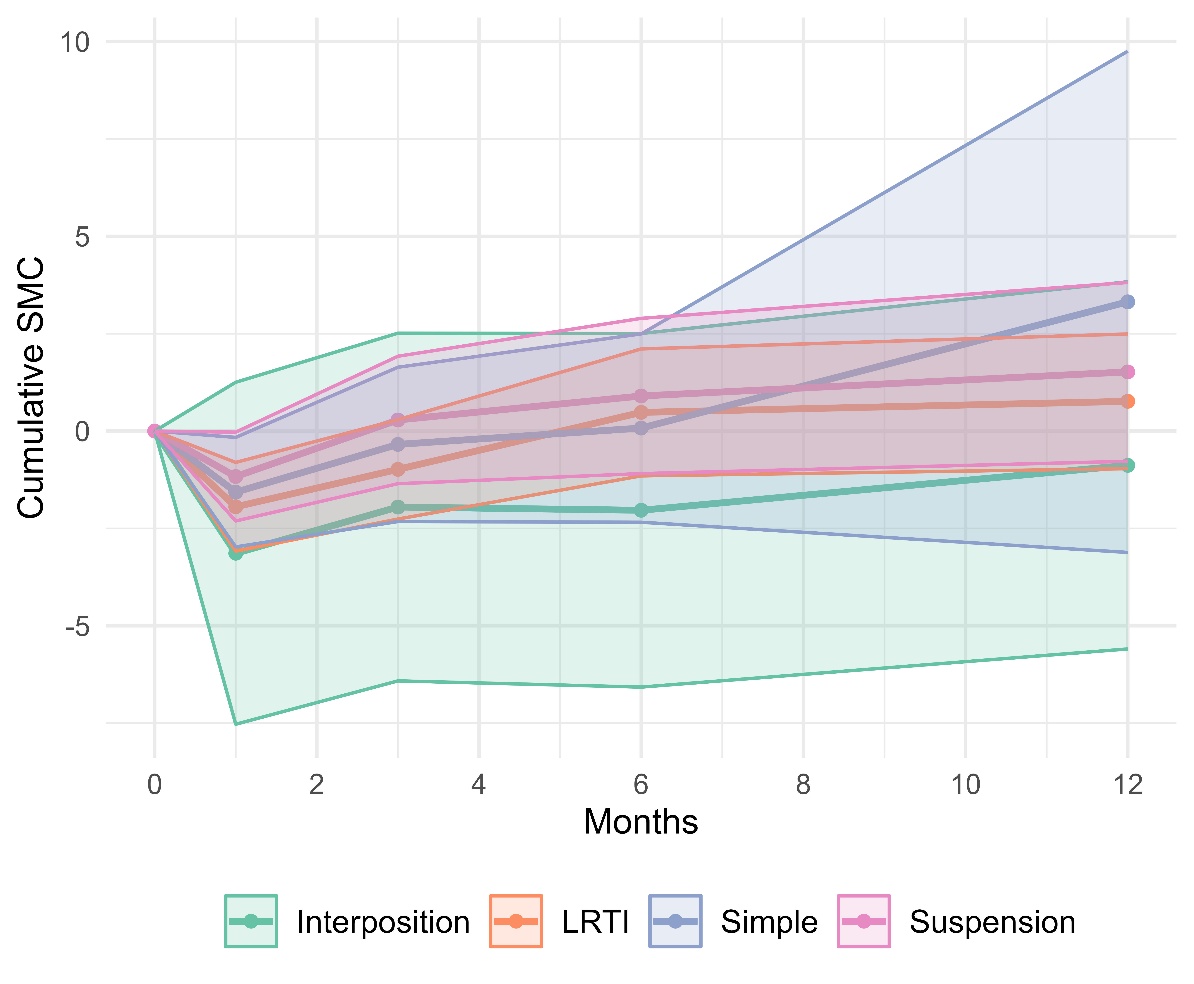

Supplement: zrag040_Supplementary_Data [file zrag040_supplementary_data.docx]
